# Genome-Wide Identification and Evolutionary Analysis of Chemosensory-Related Gene Families in the Solitary Wasp *Rhynchium brunneum* (Hymenoptera: Vespidae: Eumeninae)

**DOI:** 10.3390/insects17080764

**Published:** 2026-07-25

**Authors:** Jing Wang, Bin-Qian Li, Rui Jiang, Bin Chen, Shu-Lin He, Ting-Jing Li

**Affiliations:** Chongqing Key Laboratory of Vector Insects, Institute of Entomology and Molecular Biology, College of Life Science, Chongqing Normal University, Chongqing 401331, China; 2023110513055@stu.cqnu.edu.cn (J.W.); 2025110513011@stu.cqnu.edu.cn (B.-Q.L.); 2023051302025@stu.cqnu.edu.cn (R.J.); bin.chen@cqnu.edu.cn (B.C.)

**Keywords:** *Rhynchium brunneum*, chemosensory gene families, odorant receptors, adaptive evolution

## Abstract

The solitary predatory wasp *Rhynchium brunneum* is an important natural enemy of lepidopteran pests. In the present study, we systematically identified its chemosensory gene families, comprising 11 odorant-binding proteins (OBPs), 9 chemosensory proteins (CSPs), 147 odorant receptors (ORs), 21 gustatory receptors (GRs) and 28 ionotropic receptors (IRs). Multiple sequence alignment confirmed that OBPs and CSPs contain conserved cysteines motifs. Further alignment analysis revealed that certain genes within the GR family form high similarity clusters (up to 81%) on the same chromosome, indicative of recent tandem duplications. The IR family displays a dispersed similarity gradient (31–87%) and the OR family shows a broader spectrum (18–78%), suggesting a composite history of both recent and ancient duplication events. Phylogenetic and selection pressure analyses indicated that OBPs and CSPs are highly conserved within the Eumeninae, whereas ORs, GRs, and IRs have undergone lineage-specific expansions. All orthologous gene pairs shared with the social wasp *Vespa mandarinia* are subject to purifying selection (Ka/Ks < 1), consistent with evolutionary constraint. Numerous species-specific genes were also detected. The observed patterns suggest the hypotheses about ecological adaptations such as specific prey pheromones, location of suitable nesting sites, and so on. Resolving these relative contributions will require broader taxon sampling and functional validation assays. These results in the present study provide a genomic resource for chemosensory gene characterization in Vespidae and a foundation for future olfactory-based pest management strategies.

## Simple Summary

The solitary wasp *Rhynchium brunneum* is a natural enemy of moth and butterfly pests. We identified five chemosensory gene families in this wasp that are involved in detecting smells and tastes. Some genes are highly conserved, while others have undergone lineage-specific expansions. Compared with the social wasp *Vespa mandarinia*, all shared orthologs show signs of purifying selection, and many species-specific genes were also found. These results offer a genomic basis for understanding chemosensory evolution and future functional studies. Future studies should functionally test expanded receptors and screen active chemicals to develop attractants or repellents, which could improve the use of *R. brunneum* in integrated pest management.

## 1. Introduction

With over 5600 described species, the Vespidae plays a direct and significant role in the natural control of lepidopteran pests in agricultural, forest and orchard ecosystems. Furthermore, the family encompasses solitary, subsocial and eusocial species, rendering it a crucial model for studying the evolution of insect sociality [1,2,3]. In social hymenopterans, sophisticated olfactory communication systems underpin colony cohesion, division of labor and group defense [4]. Social vespid wasps exhibit a broad array of odor-mediated behaviors comparable to those of ants and bees. These behaviors include kin recognition, cooperative brood care, group defense and foraging [5]. Foraging workers rely not only on food-derived odors but also on intraspecific recruitment signals to enhance foraging efficiency [4,6,7]. At the molecular level, insect chemosensation relies on the coordinated action of multiple protein families, including odorant-binding proteins (OBPs), chemosensory proteins (CSPs), odorant receptors (ORs), gustatory receptors (GRs), and ionotropic receptors (IRs) [8,9]. These receptors convert chemical signals into electrical impulses that direct foraging, mating and oviposition [10,11].

Genome-wide studies have documented widespread lineage-specific expansions and contractions of chemosensory gene families in Hymenoptera. Moreover, gene duplication and positive selection act as major drivers of functional diversification [12,13]. For example, in paper wasps (*Polistes*), the 9-exon OR subfamily has undergone tandem duplications and accelerated evolution, likely linked to the perception of social signals [13]. However, current knowledge remains heavily skewed towards eusocial lineages (Vespinae and Polistinae). In striking contrast, the subfamily Eumeninae (potter wasps), the most speciose subfamily within Vespidae, representing the ancestral solitary lifestyle, remains virtually unexplored at the chemosensory molecular level [14]. Eumenine wasps are exclusively solitary predators. They rely on olfactory signals to locate prey, identify nesting materials and discriminate nest sites [15], but the underlying genetic basis is entirely unknown. 

To address this gap, we selected *Rhynchium brunneum* (Hymenoptera: Vespidae: Eumeninae), a solitary predatory wasp widely distributed across China [16]. Our laboratory has generated a high-quality, chromosome-level genome assembly of this species using PacBio and Hi-C [17]. Based on this reference genome, we performed genome-wide identification and evolutionary analysis of five major chemosensory gene families (OBP, CSP, OR, GR and IR). By systematically comparing our results with those from social Vespidae, we aim to uncover the evolutionary dynamics of chemosensory systems and to provide a molecular resource for future olfactory-based pest management strategies targeting this natural enemy. 

## 2. Materials and Methods

### 2.1. Identification of Chemosensory-Related Genes

To enable comparative analyses of chemosensory gene families, we selected one representative species from each of three subfamilies of Vespidae (Vespinae, Polistinae and Eumeninae), in addition to *Rhynchium brunneum* (Henan, China). These species were chosen to represent three distinct levels of evolution within Vespidae: the eusocial *V. mandarinia* (Vespinae) and *P. fuscatus* (Polistinae), and the solitary *O. spinipes* (Eumeninae). This sampling strategy allows for comparative analysis of chemosensory gene family evolution. Genome annotation files and proteome datasets of *Vespa mandarinia*, *Odynerus spinipes* and *Polistes fuscatus* were downloaded from the NCBI database. Hidden Markov Model (HMM) profiles are corresponding to the conserved domains of OBP (PF01395), CSP (PF03392), GR (PF08395 and PF06151), IR (PF00060) and OR (PF02949), and they were retrieved from the Pfam database (http://pfam.xfam.org/, accessed on 8 November 2025). Using these HMM profiles as queries, we conducted HMMER searches [18] against the amino acid datasets of the four target species with an E-value threshold of 1 × 10^−5^. Subsequently, all recovered candidate hits were subjected to conserved domain validation using the NCBI Conserved Domain Database (CDD; https://www.ncbi.nlm.nih.gov/cdd/, accessed on 6 December 2025) with default parameters. Sequences lacking the characteristic domains of OBPs, CSPs, GRs, IRs or ORs were discarded. Finally, CD-Hit was employed with default parameters to remove redundant protein sequences, yielding the final set of members for each chemosensory gene family. 

### 2.2. Physicochemical Property Prediction and Gene Structure Analysis of Chemosensory-Related Genes in R. Brunneum

To characterize the physicochemical properties of the chemosensory-related proteins in *R. brunneum*, we analyzed each protein sequence using the ProtParam tool on the ExPASy website (https://web.expasy.org/, accessed on 7 January 2026). The evaluated parameters included molecular weight, number of amino acids, isoelectric point, grand average of hydropathicity (GRAVY) and instability index. Subcellular localization was predicted using PSORT (https://www.genscript.com/wolf-psort.html, accessed on 15 January 2026). Gene structure information for the chemosensory-related genes was extracted from the GFF annotation file of the *R. brunneum* genome using TBtools v2.119 [19]. Based on this information, gene structure diagrams were subsequently constructed using the Gene Structure Display Server (GSDS; website (http://gsds.cbi.pku.edu.cn/, accessed on 26 January 2026).

### 2.3. Chromosomal Localization and Phylogenetic Analysis

The chromosomal distribution of chemosensory-related genes in *R. brunneum* was visualized using TBtools. To infer the phylogenetic relationships among the OBP, CSP, GR, IR, and OR genes of *V. mandarinia*, *O. spinipes*, *P. fuscatus*, and *R. brunneum*, we utilized MUSCLE for protein multisequence alignment and visualization [20], and MAFFT for multisequence alignment [21]. Phylogenetic trees were then constructed using IQ-TREE [22] with the following parameters: -m MFP (ModelFinder Plus) for automatic model selection based on the Bayesian Information Criterion (BIC), -bb 1000 for ultrafast bootstrap approximation, and -nt AUTO for automatic thread allocation. The best-fit substitution models selected by ModelFinder for each gene family were as follows: LG+I+G for OBPs, CSPs, GRs, IRs and ORs. For MAFFT, we used the L-INS-i strategy (--localpair --maxiterate 1000); for MUSCLE, we used default parameters with three iterations. CD-Hit was run with -c 0.9 (sequence identity threshold) and -n 5 (word length). In addition, we established clear criteria for branch classification. A bootstrap value of no less than 70% was set as the confidence threshold for nodes in NJ and ML phylogenetic trees. Phylogenetic units satisfying this threshold and their corresponding tree topologies were defined as major branches.

### 2.4. Selection Pressure Analysis of Chemosensory-Related Gene Families Between R. brunneum and V. mandarinia

Based on the genome annotation files of *R. brunneum* and *V. mandarinia*, the coding sequences (CDS) of five chemosensory gene families (OBP, CSP, GR, IR and OR) were extracted from both species. We selected these two species for pairwise selection pressure analysis because they are representative for solitary and eusocial wasps within Vespidae, and both of them have the most complete genome assemblies among the four species. Using the “One Step MCScanX” plugin in TBtools, synteny relationships were analyzed to identify orthologous and paralogous gene pairs, requiring at least five gene pairs per syntenic block. Tandem duplicates were defined as gene pairs located within 200 kb on the same chromosome with sequence identity ≥ 50%. “Species-specific” genes were defined as those with no syntenic ortholog detected in the *V. mandarinia* genome under these criteria. Subsequently, the “Simple Ka/Ks Calculator (NG)” tool in TBtools was employed to calculate the non-synonymous (Ka), synonymous (Ks) substitution rate, and their ratio (Ka/Ks). Ka/Ks > 1 was interpreted as consistent with positive selection, Ka/Ks = 1 with neutral evolution and Ka/Ks < 1 with purifying (negative) selection. We acknowledge, however, that such signals reflect evolutionary constraint rather than direct evidence of functional conservation, particularly in the absence of complementary functional assays. Accordingly, our interpretation remains deliberately conservative, treating Ka/Ks estimates as proxies for broad-scale selective constraints rather than as definitive indicators of gene function.

## 3. Results

### 3.1. Identification of Chemosensory-Related Gene Family Members

Based on conserved protein domains analysis, a total of five chemosensory-related gene families were identified across the four vespid species. In *R. brunneum*, 11 OBP genes, 9 CSP genes, 21 GR genes, 28 IR genes and 147 OR genes were identified. The corresponding numbers of gene family members in *V. mandarinia*, *O. spinipes* and *P. fuscatus* are summarized in Table 1. Among all families, the OR family contained the highest number of members, whereas the CSP family exhibited the lowest. Notably, the member counts for each gene family were relatively similar across the four species.

### 3.2. Physicochemical Property Prediction and Gene Structure Analysis of Chemosensory-Related Genes in R. brunneum

The physicochemical properties and gene structures of the five chemosensory gene families identified in *R. brunneum* are presented in Table S1 and Figures S1–S5, respectively.

For the OBP family, the encoded proteins ranged from 133 to 251 amino acids in length, with molecular weights of 15.42–29.46 kDa and isoelectric points (pI) ranging from 4.36 to 8.92. Hydropathicity analysis revealed that all OBPs exhibited negative GRAVY values, indicating hydrophilic properties, and subcellular localization prediction consistently placed them in the extracellular region. Similarly, the CSP family encoded proteins of 106–140 amino acids, with molecular weights of 12.44–16.08 kDa and pI values of 4.79–9.83. All CSPs also showed negative GRAVY values, confirming their hydrophilic nature, and were predicted to localize to the extracellular region. In contrast, the IR family exhibited a broader range with protein lengths ranging from 114 to 1355 amino acids, molecular weights from 12.64 to 150.96 kDa, and pI values from 4.61 to 9.67. Hydropathicity analysis revealed both positive and negative GRAVY values among IR members, indicating the presence of both hydrophilic and hydrophobic members. Most IRs were predicted to localize to the plasma membrane. The GR family encoded proteins of 139–490 amino acids, with molecular weights of 16.31–55.76 kDa and pI values of 5.43–9.64. All GRs displayed positive GRAVY values, indicating hydrophobic characteristics, and the majority were predicted to reside on the plasma membrane. Similarly, the OR family encoded proteins ranging from 78 to 1178 amino acids, with molecular weights of 8.95–136.41 kDa and pI values of 4.97–9.93. All ORs exhibited positive GRAVY values, confirming their hydrophobic proteins, and most were also predicted to localize to the plasma membrane.

The gene structures of the chemosensory-related gene families in *R. brunneum* were visualized using the GSDS website (Figures S1–S5). The results revealed that the CSP family possessed relatively simple gene structures, whereas all OBP genes contained 5–6 exons. The IR family exhibited more complex gene structures, with some members containing a large number of exons. The GR family displayed a relatively compact structure, with similar exon numbers among its members, while the OR family also displayed comparatively complex gene structures.

### 3.3. Multiple Sequence Alignment

**Figure 1:**
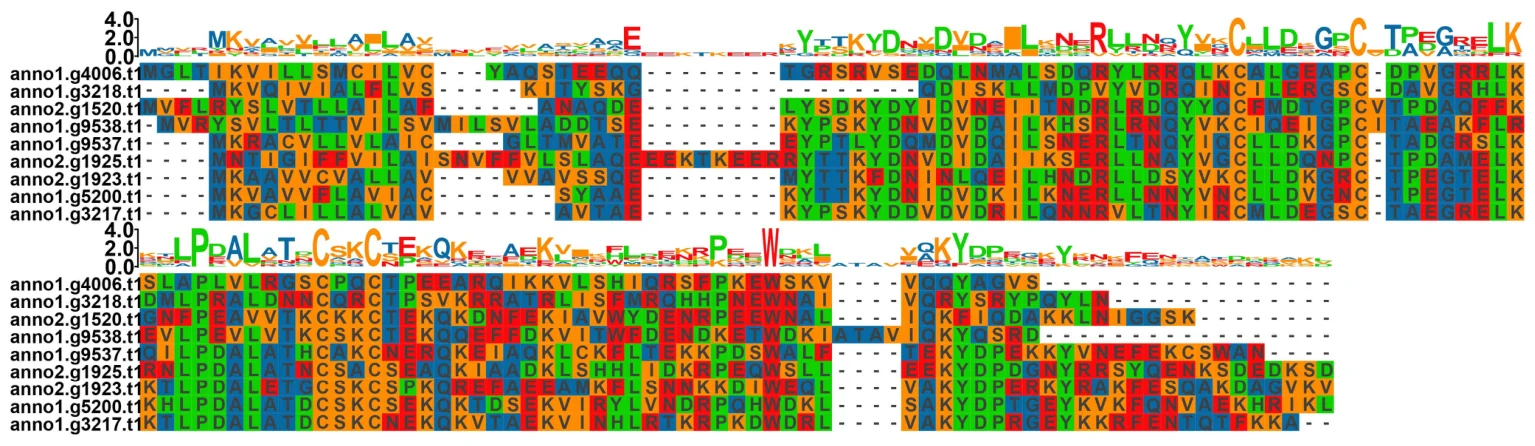
Amino acid sequences of CSPs family members in *R. brunneum*. The four conserved Cys are highlighted in yellow boxes and numbered C1–C4. The conserved motif follows the pattern C1-X_6_-C2-X_18-19_-C3-X_2_-C4. Sequence names correspond to gene IDs listed in Table S1.

**Figure 2:**
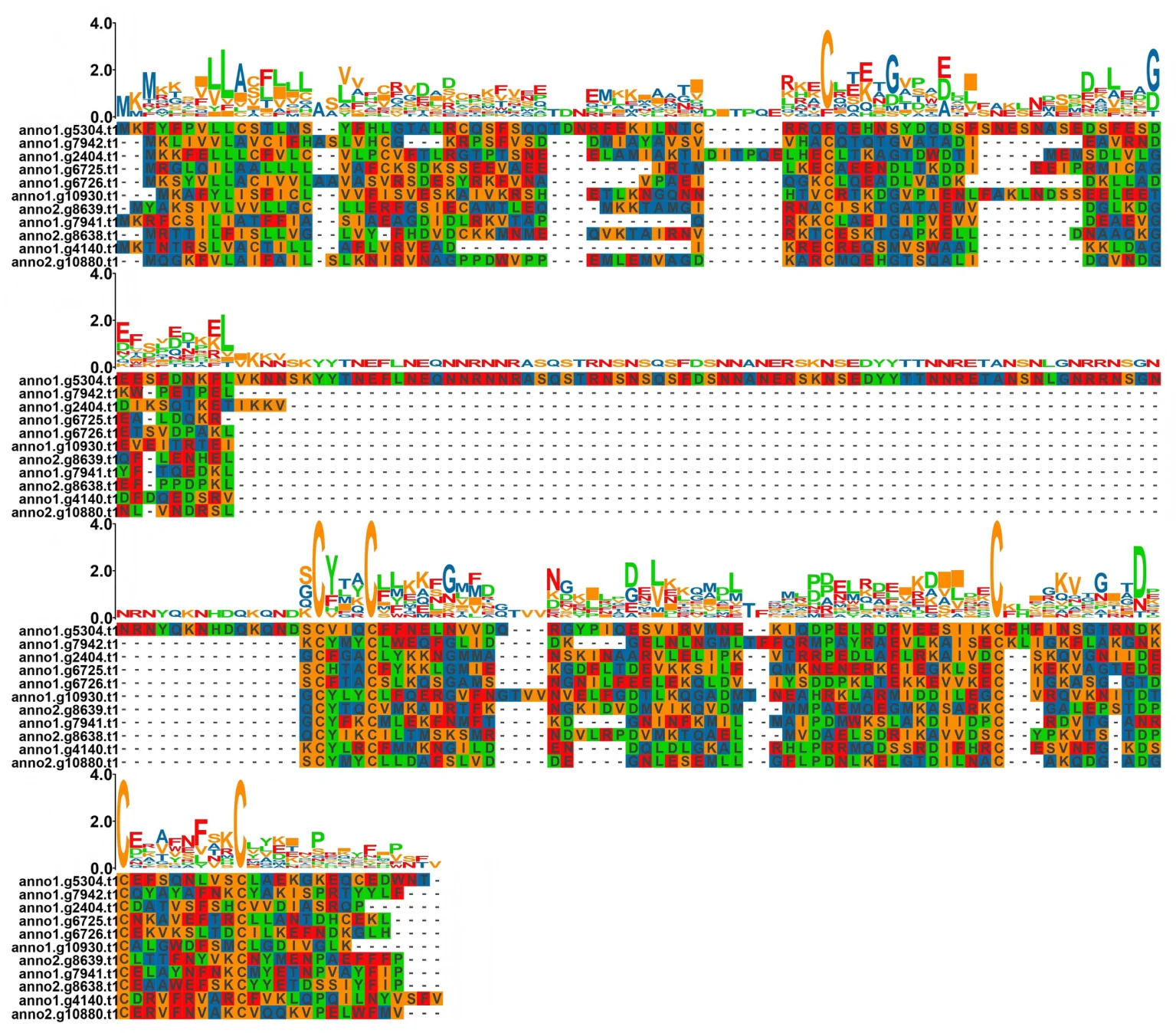
Amino acid sequences of OBPs family members in *R. brunneum*. The six conserved Cys are highlighted in yellow boxes and numbered C1–C6. The conserved motif follows the pattern C1-X_20-120_-C2-X_3_-C3-X_37-46_-C4-X_8-12_-C5-X_8_-C6. Sequence names correspond to gene IDs listed in Table S1.

The amino acid sequences of the identified chemosensory-related gene family members in *R. brunneum* are aligned using MAFFT and MUSCLE. The alignment results revealed that all CSPs contain four conserved cysteine residues (Cys), following the pattern C1-X_6_-C2-X_18-19_-C3-X_2_-C4 (Figure 1). The coefficients of variation for the intervals of both C1-C2 and C3-C4 were 0, indicating no length variability in these regions. The coefficient of variation for the C2-C3 interval was relatively low at 2.42%, suggesting limited length variability within this region in this species. The results indicate that all OBPs possess six conserved Cys, with the pattern C1-X_20-120_-C2-X_3_-C3-X_37-46_-C4-X_8-12_-C5-X_8_-C6 (Figure 2). The coefficient of variation for both C2-C3 and C5-C6 intervals were 0. In contrast, the coefficient of variation for the C1-C2 interval was notably high at 78.21%, indicating substantial length in this region. The C3-C4 interval showed a coefficient of variation at 6.64%, indicating a relatively limited range of length variation. 

**Figure 3:**
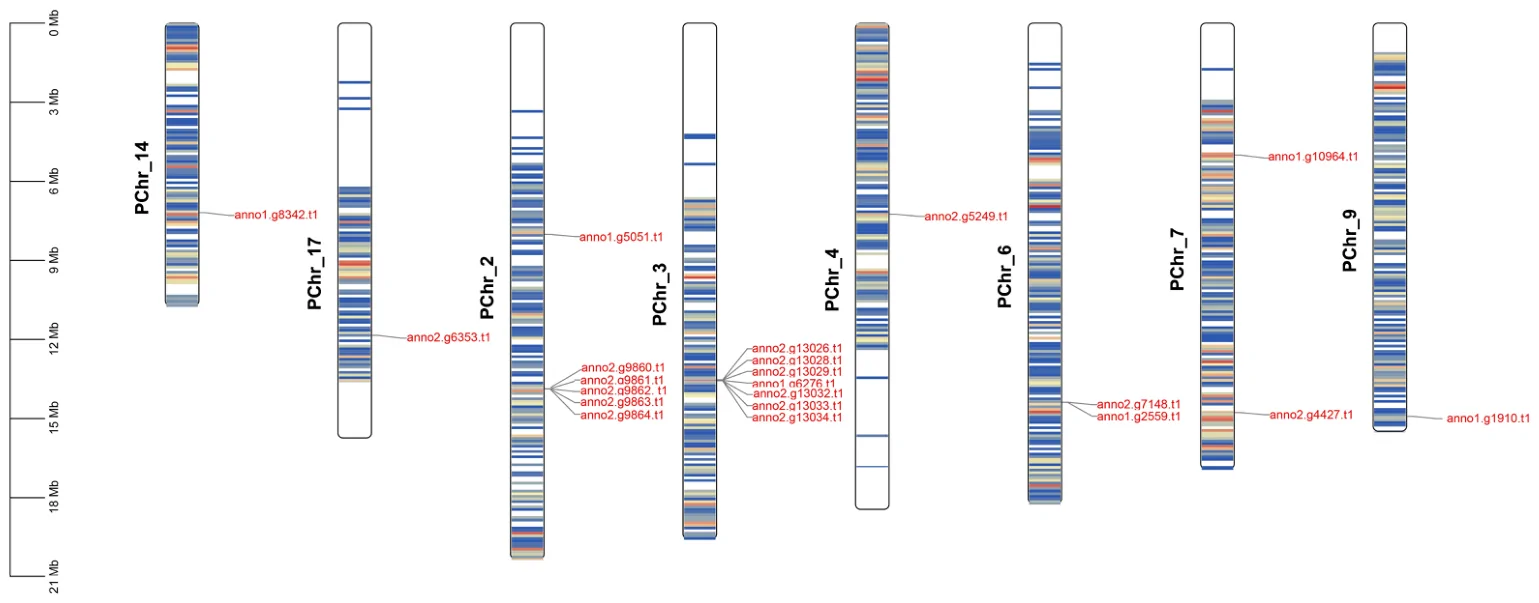
Chromosome localization analysis of the GR gene in *R. brunneum*. The eight pseudochromosomes are drawn to scale according to the chromosome-level genome assembly. Each colored rectangle represents gene density, with gene IDs indicated to the right of each pseudochromosome. The gray lines indicate the locations of genes on the pseudochromosomes; on some pseudochromosomes, multiple genes are distributed at the same position, forming tandemly arranged gene clusters.

Multiple sequence alignment revealed high similarity among certain genes within the GR gene of *R. brunneum* (Figure S6). Comparative analysis of multiple gene pairs across the three chromosomes harboring the GR family showed that several gene pairs exhibited similarity rates ranging from 51% to 81%, and all such pairs were located at identical chromosomal positions. Genes anno2.g9861.t1, anno2.g9863.t1, and anno2.g9864.t1 are located at the same position on the same chromosome with three genes in tandem (Figure 3). The highest similar values (81.06% and 79.55%) were found among a series of consecutive genes on the same chromosome (PChr-3). The concentration of 51–81% similarity pairs on PChr-2 and PChr-3 suggests that GR expansion in this solitary wasp was driven mainly by recent tandem duplication events localized to specific genomic regions (Table 2, Figure 3). This pattern is consistent with ecological adaptation, as GR genes are known to be involved in gustatory recognition of non-volatile chemicals such as nutrients and toxins [23]. The recent expansion may enhance the ability of *R. brunneum* to detect prey-specific taste signals. 

**Figure 4:**
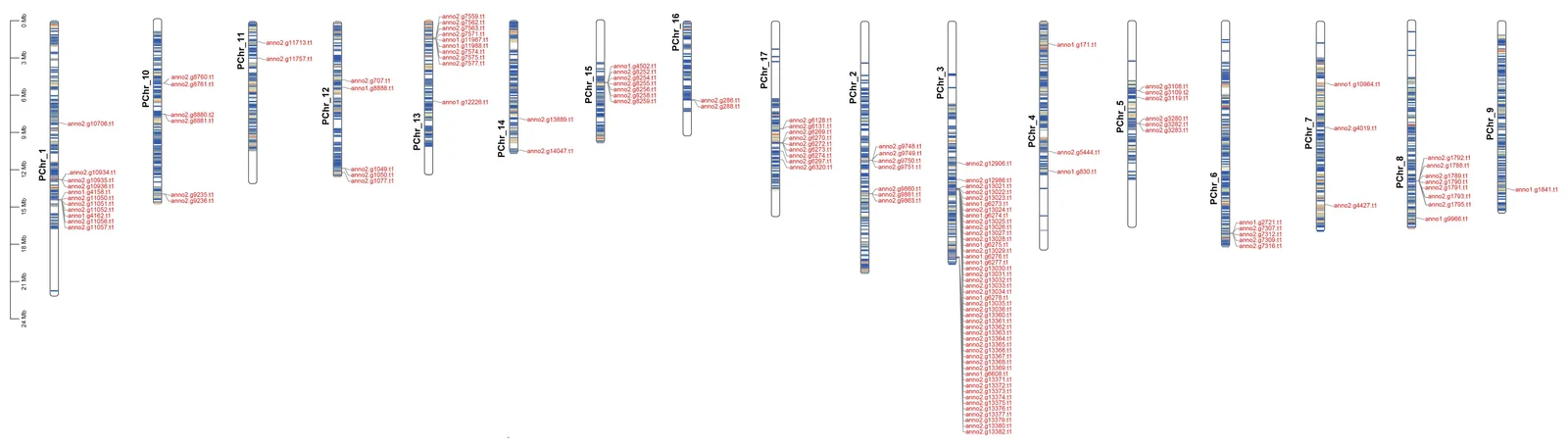
Chromosome localization analysis of the OR gene in *R. brunneum*. The seventeen pseudochromosomes are drawn to scale according to the chromosome-level genome assembly. Each colored rectangle represents gene density, with gene IDs indicated to the right of each pseudochromosome. The gray lines indicate the locations of genes on the pseudochromosomes; on some pseudochromosomes, multiple genes are distributed at the same position, forming tandemly arranged gene clusters.

In contrast, the IR family displayed pronounced variation in gene similarity (Figure S7). Sequence similarity among multiple gene pairs distributed across the four chromosomes revealed substantial differences, particularly for two gene pairs on chromosome PChr-11, where similarity ranged from approximately 30% to nearly 80%. Chromosomal mapping (Figure S11) indicated that numerous gene pairs on chromosome PChr-10 showed similarities between 40% and 60%. Notably, one gene pair on chromosome PChr-8 exhibited the highest sequence similarity among IRs, at approximately 90% (Table 3). Corresponding, the OR family (Figure S8) exhibited a broad range of sequence similarity, from 18% to 78%. Anno2.g13032.t1, anno2.g13033.t1, anno1.g6276.t1, anno2.g13026.t1, anno2.g13028.t1 and anno2.g13029.t1 share identical chromosomal positions. Furthermore, there is a high degree of similarity among the members, suggesting that they may constitute a typical tandemly arranged gene cluster (Table 4, Figure 4). Chromosomal localization analysis (Figure 4) revealed no significant differences in similarity between gene pairs at identical positions on the same chromosome (PChr-5, PChr-17; Table 4). 

### 3.4. Chromosomal Localization and Phylogenetic Analysis

To investigate the evolutionary relationships of chemosensory-related gene families in *R. brunneum*, phylogenetic trees were constructed using gene sequences from *R. brunneum*, *O. spinipes*, *V. mandarinia* and *P. fuscatus*. The OBP-based phylogenetic tree (Figure S12) showed that the OBPs of *R. brunneum* are distributed across three major clades. Most of the *R. brunneum* OBP genes clustered with those of *O. spinipes*, reflecting sequence conservation within the subfamily Eumeninae. Similarly, the CSP-based phylogenetic tree (Figure S13) also revealed a close clustering between *R. brunneum* and *O. spinipes*, further supporting conservation within Eumeninae. 

**Figure 5:**
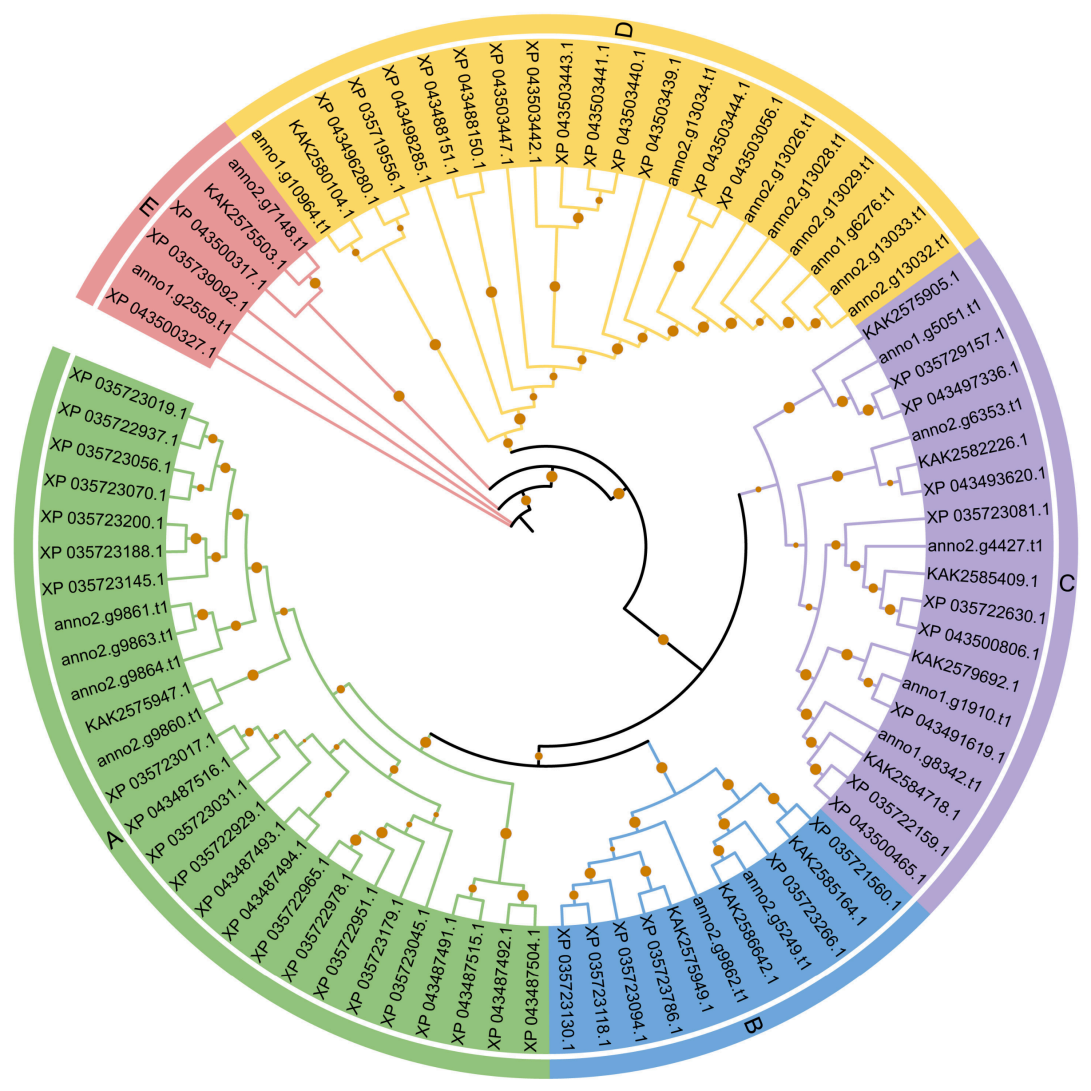
Phylogenetic relationships of GRs among *R. brunneum*, *O. spinipes*, *V. mandarinia* and *P. fuscatus* (anno: *R. brunneum*; KAK: *O. spinipes*; XP043: *P. fuscatus*; XP035: *V. mandarinia*). Based on the tree’s topology and node support values, the GR proteins from four species were classified into five major branches, each distinguished by a different color.

In the GR-based phylogenetic tree (Figure 5), several GR genes of *R. brunneum* formed monophyletic clades within clades A and D. Chromosomal localization analysis (Figure 4), revealed that genes anno2.g9861.t1, anno2.g9863.t1, and anno2.g9864.t1 genes are located at the same position on the same chromosome. Likewise, anno2.g13032.t1, anno2.g13033.t1, anno1.g6276.t1, anno2.g13026.t1, anno2.g13028.t1 and anno2.g13029.t1 share identical chromosomal positions. These findings indicate that gene duplication events have occurred in clades A and D, suggesting a possible species-specific expansion of the GR gene family. The expansion of GR genes in *R. brunneum* may be associated with its need as a solitary predator to recognize taste signals from specific lepidopteran prey, consistent with observations that GR expansion in insects correlates with dietary adaptation [24]. Similar patterns were observed in *V. mandarinia* and *P. fuscatus*. Notably, clade A was predominantly composed of GR genes from the social species *V. mandarinia* and *P. fuscatus*, whereas GR genes from the solitary species *R. brunneum* and *O. spinipes* were less abundant in this clade. GR genes from the solitary species *O. spinipes* and the social species *V. mandarinia* were less abundant in clade D.

In the IR-based phylogenetic tree (Figure S14), a possible gene duplication event was also detected for IR genes of *V. mandarinia* in clade A. The IR genes of *V. mandarinia* in the remaining clades, as well as those of *R. brunneum* across all clades, exhibit sequence conservation within the Vespidae family. 

**Figure 6:**
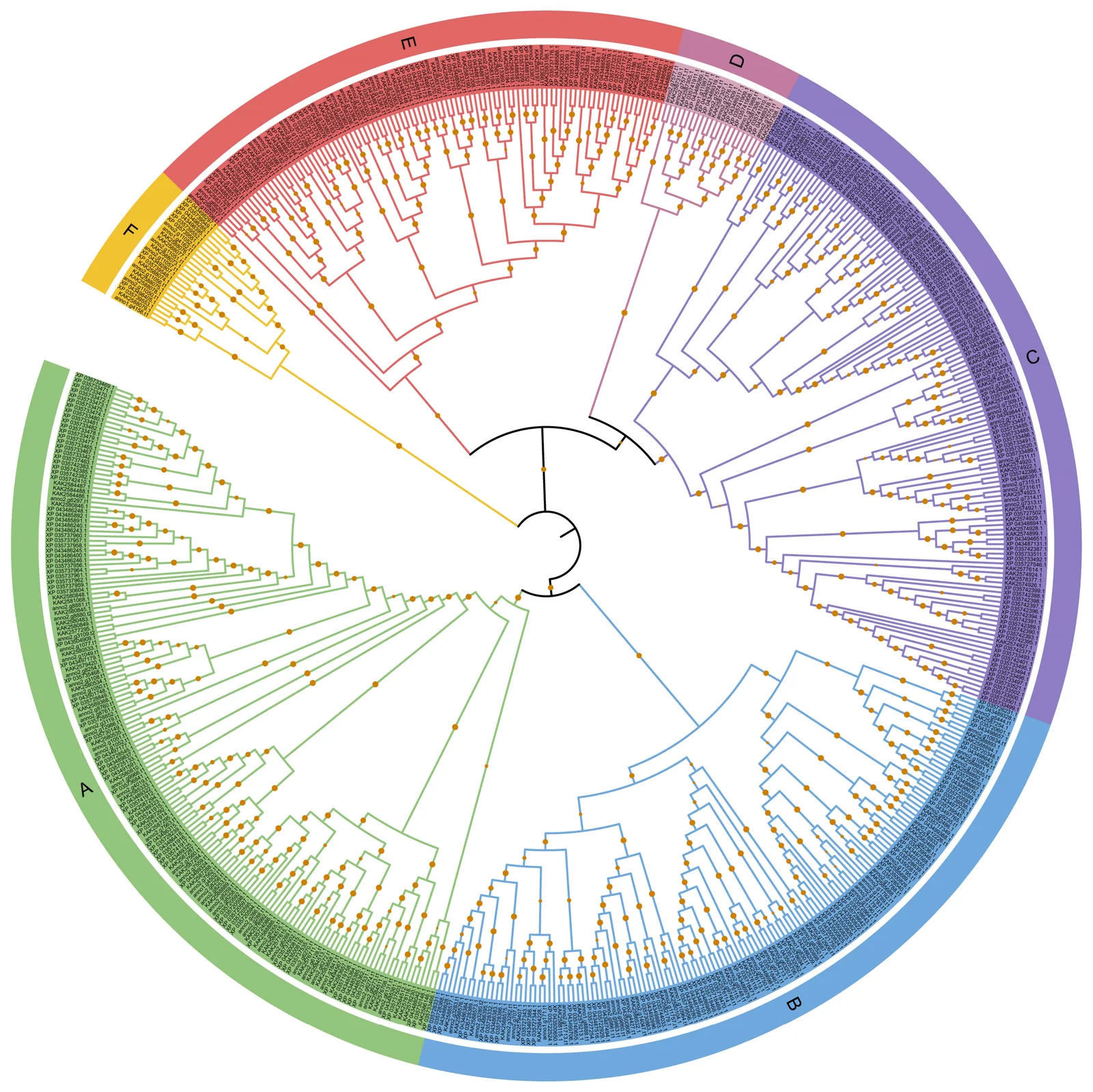
Phylogenetic relationships of ORs among *R. brunneum*, *O. spinipes*, *V. mandarinia* and *P. fuscatus* (anno: *R. brunneum*; KAK: *O. spinipes*; XP043: *P. fuscatus*; XP035: *V. mandarinia*). Based on the tree’s topology and node support values, the OR proteins from four species were classified into six major branches, each distinguished by a different color.

In the OR-based phylogenetic tree (Figure 6), some OR genes of *R. brunneum* clustered with those of *V. mandarinia* or *P. fuscatus*, suggesting that these genes may have originated from a common ancestor of Vespidae. Furthermore, monophyletic clades formed by OR genes from *R. brunneum* were observed in clades A, B and C. Chromosomal localization analysis (Figure 6), that genes anno1.g4502.t1, anno2.g8256.t1, and anno2.g8253.t1 are located at the same position on the same chromosome, as are anno2.g1790.t1, anno2.g1791.t1, anno2.g1793.t1, anno2.g1789.t1, and anno2.g1792.t1. These results indicate that gene duplication events likely also occurred in the OR gene family. Additionally, chromosomal localization maps for the OBP, CSP, and IR families are provided in the appendix (Figure S9–S11). A previous study on paper wasp OR genes also reported that the 9-exon OR subfamily underwent lineage-specific tandem duplication and may be under positive selection [13], indicating that OR expansion is a recurring theme in vespid evolution. 

### 3.5. Selection Pressure Analysis

**Figure 7:**
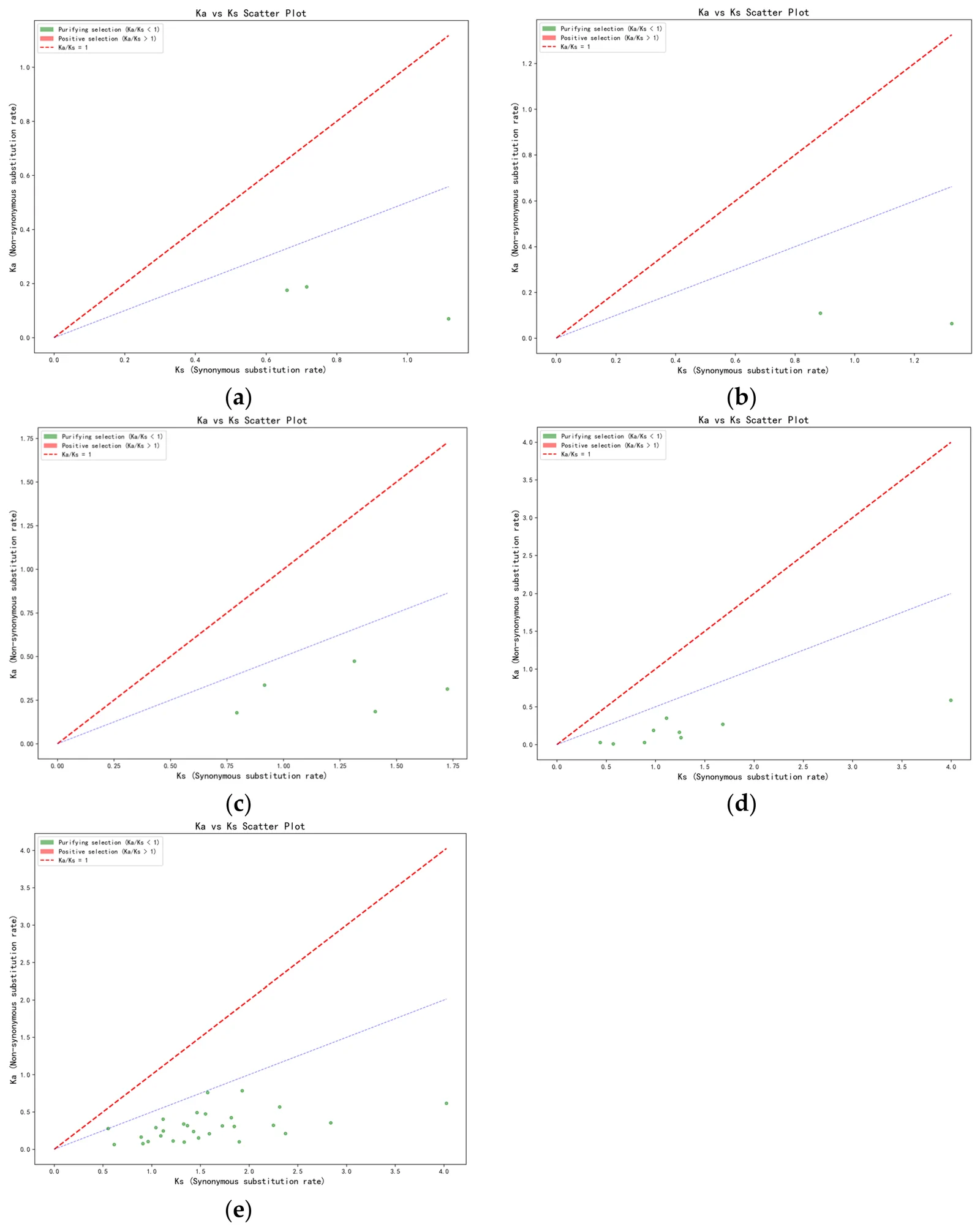
Ka/Ks analysis of orthologous OBP, CSP, GR, IR and OR gene pairs between *R. brunneum* and *V. mandarinia*: (**a**) Ka/Ks analysis of orthologous OBP gene pairs between *R. brunneum* and *V. mandarinia*; (**b**) Ka/Ks analysis of orthologous CSP gene pairs between *R. brunneum* and *V. mandarinia*; (**c**) Ka/Ks analysis of orthologous GR gene pairs between *R. brunneum* and *V. mandarinia*; (**d**) Ka/Ks analysis of orthologous IR gene pairs between *R. brunneum* and *V. mandarinia*; (**e**) Ka/Ks analysis of orthologous OR gene pairs between *R. brunneum* and *V. mandarinia*. Each green point represents one orthologous gene pair identified through synteny analysis using MCScanX.

Based on synteny analysis between the genomes of *R. brunneum* and *V. mandarinia*, we identified orthologous gene pairs in each chemosensory gene family and calculated their Ka/Ks ratios to infer selection pressures. In the OBP family, three orthologous pairs were identified, all with Ka/Ks values less than 1 (Figure 7a). The remaining eight OBP genes were specific to *R. brunneum*. In the CSP gene family, two orthologous pairs were found, also with Ka/Ks less than 1 (Figure 7b); the other seven CSP genes were species-specific. In the GR family, five orthologous pairs exhibited Ka/Ks values less than 1 (Figure 7c), and the remaining 16 GR genes were specific to *R. brunneum*. For the IR family, nine orthologous pairs showed Ka/Ks values less than 1 (Figure 7d), with the other 19 IR genes being species-specific. Finally, in the OR family, 29 orthologous pairs had Ka/Ks values less than 1 (Figure 7e); the remaining 118 OR genes were unique to *R. brunneum*. The presence of many species-specific genes (118 OR, 16 GR, and 19 IR genes) with no orthologs detected in *V. mandarinia*.

## 4. Discussion

Across four vespid species, the total chemosensory gene counts varied noticeably, with social species (e.g., *V. mandarinia*) possessing larger repertoires than solitary ones, particularly for ORs and GRs. This quantitative divergence supports the notion that sociality imposes additional chemosensory demands [7]. 

OBPs and CSPs are conserved in both number and structure. Across the four vespid species, the counts of OBP and CSP counts remain remarkably stable, with 11 and 9 members, respectively, supporting their role as conserved scaffolds that undergo slow turnover [11]. The conserved cysteine motifs, namely six Cys in OBPs and the canonical C1-X_6_-C2-X_18-19_-C3-X_2_-C4 in CSPs, are identical to those in other hymenopterans, affirming their structural conservation. In our study, OBPs and CSPs were identified as hydrophilic proteins localized to the extracellular region, consistent with their function of transporting hydrophobic odorant within the sensillum lymph. Collectively, these findings confirm the high structural and functional stability of soluble carriers in this lineage. Nevertheless, despite this stasis, no lineage-specific expansions or contractions were observed, suggesting that the evolution of soluble carriers is decoupled from the rapid diversification of transmembrane receptors in this lineage. 

In *R. brunneum*, GR expansion appears both localized and recent. The GR genes are concentrated on PChr-2 and PChr-3, where pairs with 60–81% sequence similarity reside almost exclusively on the same chromosome. The highest-similarity pairs (81.06% and 79.55%) map to PChr-3 and likely arose from a single, recent tandem duplication event, as evidenced by their adjacent chromosomal positions and the lack of intervening unrelated genes. This arrangement is consistent with the “birth-and-death” model documented in other Hymenoptera [25,26]. However, the restricted genomic location contrasts with the more dispersed GR expansions reported in polyphagous species. Previous studies have associated GR gene expansion with species adaptation to specific ecological niches [27]. Given that GRs are primarily responsible for detecting non-volatile chemical substances, including nutrients, toxins, and pheromones, thereby influencing feeding preference and oviposition behavior [28], we tentatively suggest that this localized expansion may reflect adaptation to specific prey-derived gustatory cues.

In *R. brunneum*, OR and IR families exhibit distinct duplication chronologies. OR genes show a wide similarity spectrum (18–78%). High-similarity pairs (≥75%) are located on PChr-8 and PChr-3. They are often found in tandem arrays (e.g., anno2.g1790.t1–1791.t1), indicating recent duplications. Low-similarity pairs (<30%) likely represent ancient duplications or distinct subfamilies, pointing to a multi-phase expansion history. This layered pattern is reminiscent of OR evolution in ants and bees [29,30]. Given that ORs mediate volatile chemical detection critical for host location, mate choice, and oviposition site selection [10], the complex expansion history in *R. brunneum* likely reflects adaptation to the diverse olfactory environments encountered during independent foraging and nesting activities. By contrast, IR genes display a more continuous similarity gradient (31–87%), without obvious hotspots for recent duplication. This more uniform distribution of sequence divergence might be explained by their broader functional repertoire. This repertoire includes temperature and humidity sensing in addition to chemoreception [31]. Functionally, IRs serve as complementary chemosensors and, together with ORs and GRs, constitute the peripheral chemosensory apparatus of insects. Moreover, all four vespid species possess substantially more IRs than fig wasps (5–12 per species) [32], suggesting that vespids face a wider array of chemosensory challenges. 

Phylogenetic clustering and selection patterns reveal both conservation and lineage-specific innovation. In the GR tree, several monophyletic clades comprising only *R. brunneum* genes indicate independent duplications after the split from social vespids. This may reflect dietary adaptation as seen in aphids where GR expansion is linked to host range shifts [33]. Similarly, the OR tree shows that some of *R. brunneum* ORs cluster with orthologs from *V. mandarinia* or *P. fuscatus*, reflecting ancestral vespid repertoires. Others form species-specific clades, likely derived from recent tandem events. Ka/Ks analysis of all orthologous pairs across species yielded values far below 1, confirming strong purifying selection on shared chemosensory genes. The result is consistent with evolutionary constraint and may reflect the maintenance of essential functions [34]. Strikingly, a substantial number of *R. brunneum* chemosensory genes lack detectable orthologs in *V. mandarinia*, implying rapid lineage-specific turnover. This observation is consistent with the birth-and-death model of chemoreceptor evolution [26], where gene duplication provides raw material for adaptive diversification. For the solitary *R. brunneum*, these newly evolved chemosensory receptor genes may facilitate the recognition of specific prey pheromones, location of suitable nesting sites, or perception of unique habitat chemical signatures. But we are currently unable to directly test the functional consequences of these genomic changes.

## 5. Conclusions

In this study, we identified the chromosome-level genome of the solitary wasp *Rhynchium brunneum*. Specifically, we found 147 ORs, 28 IRs, 21 GRs, 11 OBPs, and 9 CSPs. OBPs and CSPs are conserved in both cysteine motifs and gene numbers across vespid species. In contrast, ORs, GRs, and IRs have undergone lineage-specific tandem duplications with distinct patterns. GRs show localized and recent expansions, ORs display a multi-phase history of both recent and ancient events, and IRs exhibit a more continuous divergence gradient. Orthologous genes shared with the social wasp *V. mandarinia* are under purifying selection (Ka/Ks < 1), consistent with evolutionary constraint rather than direct evidence of functional conservation. In addition, numerous species-specific genes indicate rapid turnover. We compared solitary and social vespid species, but with only four species representing three different subfamilies, we cannot formally disentangle the effects of sociality from those of phylogenetic history or ecological niche and so on. Broader taxon sampling and functional assays are required to resolve these contributions. This work supplies a genomic data and hypotheses for future studies on solitary and eusocial vespids, and serve as a foundation for developing olfactory-based pest management. Further studies including transcriptomic, electrophysiological, and behavioral studies will be necessary to validate receptor functions and test ecological hypotheses in future. 

## Figures and Tables

**Table 1:** Number of chemosensory-related genes identified in different Vespidae species.

Species	OBP	CSP	GR	IR	OR
*R. brunneum*	11	9	21	28	147
*O. spinipes*	13	11	11	35	132
*P. fuscatus*	8	7	26	32	121
*V. mandarinia*	10	9	27	38	178

**Table 2:** GR amino acid sequence alignment table of *R. brunneum*.

Chromosome	Gene1	Gene2	Sequence Similarity
PChr-6	anno2.g7148.t1	anno1.g2559.t1	62.07%
PChr-2	anno2.g9860.t1 anno2.g9863.t1	anno2.g9861.t1 anno2.g9861.t1	51.26% 69.11%
PChr-3	anno2.g13034.t1 anno2.g13034.t1 anno2.g13034.t1 anno2.g13034.t1 anno2.g13034.t1 anno2.g13034.t1 anno2.g13032.t1 anno2.g13029.t1 anno2.g13029.t1	anno2.g13029.t1 anno2.g13032.t1 anno1.g6276.t1 anno2.g13026.t1 anno2.g13028.t1 anno2.g13033.t1 anno2.g6276.t1 anno2.g6276.t1 anno2.g13028.t1	59.85% 63.64% 63.34% 64.39% 65.15% 65.91% 79.55% 79.55% 81.06%

**Table 3:** IR amino acid sequence alignment table of *R. brunneum*.

Chromosome	Gene1	Gene2	Sequence Similarity
PChr-11	anno1.g10140.t1 anno1.g10260.t2	anno1.g10141.t3 anno2.g11841.t1	31.25% 81.75%
PChr-9	anno2.g2090.t1	anno2.g2091.t1	35.98%
PChr-10	anno2.g9190.t2 anno1.g7154.t1 anno1.g6858.t1 anno1.g6858.t1 anno1.g6857.t2 anno1.g6857.t2	anno2.g9191.t1 anno2.g9077.t1 anno1.g6855.t1 anno1.g6859.t1 anno1.g6859.t1 anno1.g6858.t1	42.32% 44.74% 53.38% 58.58% 59.58% 61.19%
PChr-8	anno2.g1521.t1	anno2.g1525.t1	87.27%

**Table 4:** OR amino acid sequence alignment table of *R. brunneum*.

Chromosome	Gene1	Gene2	Sequence Similarity
PChr-2	anno2.g9748.t1 anno2.g9750.t1	anno2.g9749.t1 anno2.g9751.t1	18.49% 42.04%
PChr-1	anno1.g4158.t1 anno1.g4158.t1 anno2.g10934.t1 anno2.g10934.t1	anno2.g11051.t1 anno2.g11050.t1 anno2.g10936.t1 anno2.g10935.t1	20.15% 28.45% 51.26% 58.82%
PChr-10	anno2.g8760.t1 anno2.g8880.t2	anno2.g8761.t1 anno2.g8881.t2	20.18% 33.33%
PChr-17	anno2.g6272.t1 anno2.g6272.t1	anno2.g6270.t1 anno2.g6269.t1	28.95% 32.48%
PChr-5	anno2.g3283.t1 anno2.g3283.t1	anno2.g3280.t1 anno2.g3282.t1	39.17% 44.44%
PChr-13	anno1.g11987.t1 anno2.g7559.t1	anno1.g11988.t1 anno2.g7562.t1	38.14% 50.86%
PChr-8	anno2.g1790.t1	anno2.g1791.t1	78.07%
PChr-3	anno2.g13021.t1 anno2.g13368.t1	anno2.g13022.t1 anno2.g13369.t1	51.75% 74.79%

## Data Availability

The data presented in this study are openly available from the National Center for Biotechnology Information at https://www.ncbi.nlm.nih.gov, accessed on 16 March 2025. The reference genome of the study species *R. brunneum* was sequenced by our laboratory and is available from https://identifiers.org/insdc.gca:GCA_055773155.1, accessed on 6 April 2026 [17].

## Supplementary Materials

The following supporting information can be downloaded at: https://www.mdpi.com/article/10.3390/insects17080764/s1, Figure S1: Gene structure of CSP family members in *R. brunneum*. Figure S2: Gene structure of OBP family members in *R. brunneum*; Figure S3: Gene structure of GR family members in *R. brunneum*; Figure S4: Gene structure of IR family members in *R. brunneum*; Figure S5: Gene structure of OR family members in *R. brunneum*; Figure S6: Amino acid sequences of GRs family members in *R. brunneum*; Figure S7: Amino acid sequences of IRs family members in *R. brunneum*; Figure S8: Amino acid sequences of ORs family members in *R. brunneum*; Figure S9: Chromosome localization analysis of the CSP gene in *R. brunneum*; Figure S10: Chromosome localization analysis of the OBP gene in *R. brunneum*; Figure S11: Chromosome localization analysis of the IR gene in *R. brunneu*; Figure S12: Phylogenetic relationships of OBPs among *R. brunneum*, *O. spinipes*, *V. mandarinia* and *P. fuscatus* (anno: *R. brunneum*; KAK: *O. spinipes*; XP043: *P. fuscatus*; XP035: *V. mandarinia*); Figure S13: Phylogenetic relationships of CSPs among *R. brunneum*, *O. spinipes*, *V. mandarinia* and *P. fuscatus* (anno: *R. brunneum*; KAK: *O. spinipes*; XP043: *P. fuscatus*; XP035: *V. mandarinia*); Figure S14: Phylogenetic relationships of IRs among *R. brunneum*, *O. spinipes*, *V. mandarinia* and *P. fuscatus* (anno: *R. brunneum*; KAK: *O. spinipes*; XP043: *P. fuscatus*; XP035: *V. mandarinia*); Table S1: Summary of physicochemical properties of CSP, OBP, OR, IR, and GR gene families in *R. brunneum*.

## References

[1] Hunt J.H. (2007). The Evolution of Social Wasps.

[2] Pickett K.M., Carpenter J.M. (2010). Simultaneous analysis and the origin of eusociality in the Vespidae (Insecta: Hymenoptera). Arthropod Syst. Phylogeny.

[3] Blaimer B.B., Santos B.F., Cruaud A., Gates M.W., Kula R.R., Mikó I., Rasplus J.-Y., Smith D.R., Talamas E.J., Brady S.G. (2023). Key innovations and the diversification of Hymenoptera. Nat. Commun..

[4] Couto A., Lapeyre B., Thiéry D., Sandoz J.-C. (2016). Olfactory pathway of the hornet *Vespa velutina*: New insights into the evolution of the hymenopteran antennal lobe. J. Comp. Neurol..

[5] Edwards R. (1980). Social Wasps, Their Biology and Control.

[6] Ono M., Igarashi T., Ohno E., Sasaki M. (1995). Unusual thermal defence by a honeybee against mass attack by hornets. Nature.

[7] Ono M., Terabe H., Hori H., Sasaki M. (2003). Components of giant hornet alarm pheromone. Nature.

[8] Kordaczuk J., Wojda I. (2026). Insect olfactory proteins: A comprehensive review with a special emphasis on the role of odorant-binding proteins in insect immunity. Insect Sci..

[9] Abendroth J.A., Moural T.W., Wei H., Zhu F. (2023). Roles of insect odorant binding proteins in communication and xenobiotic adaptation. Front. Insect Sci..

[10] Zhou X., Rokas A., Berger S.L., Liebig J., Ray A., Zwiebel L.J. (2015). Chemoreceptor evolution in Hymenoptera and its implications for the evolution of eusociality. Genome Biol. Evol..

[11] Sánchez-Gracia A., Vieira F.G., Rozas J. (2009). Molecular evolution of the major chemosensory gene families in insects. Heredity.

[12] Zhou X., Slone J.D., Rokas A., Berger S.L., Liebig J., Ray A., Reinberg D., Zwiebel L.J. (2012). Phylogenetic and transcriptomic analysis of chemosensory receptors in a pair of divergent ant species reveals sex-specific signatures of odor coding. PLoS Genet..

[13] Legan A., Jernigan C., Miller S., Fuchs M., Sheehan M. (2021). Expansion and accelerated evolution of 9-exon odorant receptors in *Polistes* paper wasps. Mol. Biol. Evol..

[14] Cowan D.P. (2018). The Social Biology of Wasps.

[15] Dang H.T., Nguyen L.T.P. (2019). Nesting biology of the potter wasp *Rhynchium brunneum brunneum* (Fabricius, 1793) (Hymenoptera: Vespidae: Eumeninae) in North Vietnam. J. Asia-Pac. Entomol..

[16] Peng Y.L., He S.L., Chen B., Li T.J. (2025). An integrative phylogenetic analysis of the genus *Rhynchium* Spinola (Hymenoptera: Vespidae: Eumeninae) from China based on morphology, genomic data and geographical distribution. Insects.

[17] Wang J., He S.L., Chen B., Li T.J. (2026). Chromosome-level genome assembly of *Rhynchium brunneum* (Fabricius, 1793) (Hymenoptera: Vespidae). Sci. Data.

[18] Eddy S.R. (2011). Accelerated profile hmm searches. PLoS Comput. Biol..

[19] Chen C., Chen H., Zhang Y., Thomas H.R., Frank M.H., He Y., Xia R. (2020). Tbtools: An integrative toolkit developed for interactive analyses of big biological data. Mol. Plant.

[20] Edgar R.C. (2004). Muscle: Multiple sequence alignment with high accuracy and high throughput. Nucleic Acids Res..

[21] Katoh K., Standley D.M. (2013). Mafft multiple sequence alignment software version 7: Improvements in performance and usability. Mol. Biol. Evol..

[22] Minh B.Q., Schmidt H.A., Chernomor O., Schrempf D., Woodhams M.D., von Haeseler A., Lanfear R. (2020). Iq-tree 2: New models and efficient methods for phylogenetic inference in the genomic era. Mol. Biol. Evol..

[23] Slone J., Daniels J., Amrein H. (2007). Sugar receptors in Drosophila. Curr. Biol..

[24] Vertacnik K.L., Herrig D.K., Godfrey R.K., Hill T., Geib S.M., Unckless R.L., Nelson D.R., Linnen C.R. (2021). Ecological correlates of gene family size in a pine-feeding sawfly genome and across Hymenoptera. Mol. Biol. Evol..

[25] McKenzie S.K., Kronauer D.J.C. (2018). The genomic architecture and molecular evolution of ant odorant receptors. Genome Res..

[26] Nei M., Rooney A.P. (2005). Concerted and birth-and-death evolution of multigene families. Annu. Rev. Genet..

[27] Suzuki H.C., Ozaki K., Makino T., Uchiyama H., Yajima S., Kawata M. (2018). Evolution of gustatory receptor gene family provides insights into adaptation to diverse host plants in nymphalid butterflies. Genome Biol. Evol..

[28] Pentzold S., Burse A., Boland W. (2017). Contact chemosensation of phytochemicals by insect herbivores. Nat. Prod. Rep..

[29] Zhang B., Yang R.R., Jiang X.C., Xu X.X., Wang B., Wang G.R. (2023). Genome-wide analysis of the odorant receptor gene family in *Solenopsis invicta*, *Ooceraea biroi*, and *Monomorium pharaonis* (Hymenoptera: Formicidae). Int. J. Mol. Sci..

[30] Tom M.T., Brand P., Bucks S., Zhang J., Escobar Huezo M.E., Hansson B.S., Bisch-Knaden S. (2024). Gene expansion in the hawkmoth *Manduca sexta* drives evolution of food-associated odorant receptors. iScience.

[31] Eyun S.I., Soh H.Y., Posavi M., Munro J.B., Hughes D.S., Murali S.C., Qu J., Dugan S., Lee S.L., Chao H. (2017). Evolutionary history of chemosensory-related gene families across the Arthropoda. Mol. Biol. Evol..

[32] Yu H., Nong X., Huang W., Yang L., Bhanumas C., Xiong Y., Dai S. (2025). Ionotropic receptor genes in fig wasps: Evolutionary insights from comparative studies. Insects.

[33] Olvera-Vazquez S.G., Chen X., Mesnil A., Meslin C., Almeida-Silva F., Confais J., Bourgeois Y., Lombardi G., Lougmani C., Alix K. (2025). Comprehensive annotation of olfactory and gustatory receptor genes and transposable elements revealed their evolutionary dynamics in aphids. Mol. Biol. Evol..

[34] Yu H., Nong X., Fan S., Bhanumas C., Deng X., Wang R., Chen X., Compton S.G. (2023). Olfactory and gustatory receptor genes in fig wasps: Evolutionary insights from comparative studies. Gene.

