# Supplementary material for: Genome-Wide Identification and Evolutionary Analysis of Chemosensory-Related Gene Families in the Solitary Wasp Rhynchium brunneum (Hymenoptera: Vespidae: Eumeninae)
Source: Insects. 2026 Jul 25;17(8):764. doi: 10.3390/insects17080764 (PMC13513145; doi:10.3390/insects17080764)
Supplement: Supplementary file 1 [file insects-17-00764-s001.zip › insects-4381331-supplementary.pdf]

Supplementary Materials

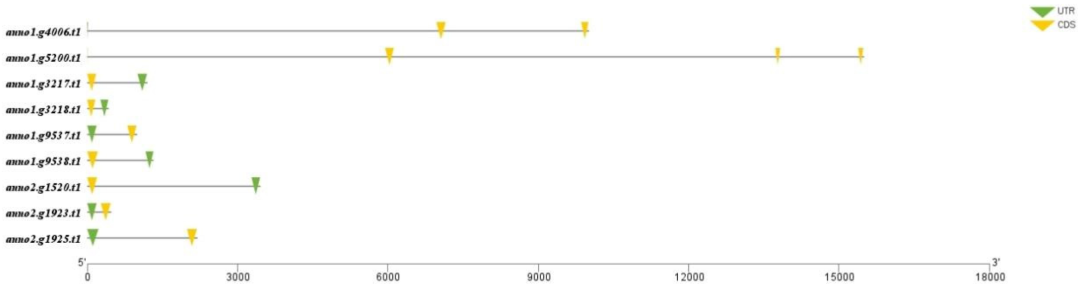

Supplementary Figure S1. Gene structure of CSP family members in *R. brunneum.* Exons are represented by yellow boxes, introns by black lines, and untranslated regions (UTRs) by green boxes. Gene IDs are shown on the left. The scale bar at the bottom indicates the length of the gene (in bp). All CSP genes exhibit relatively simple structures with 2–3 exons.

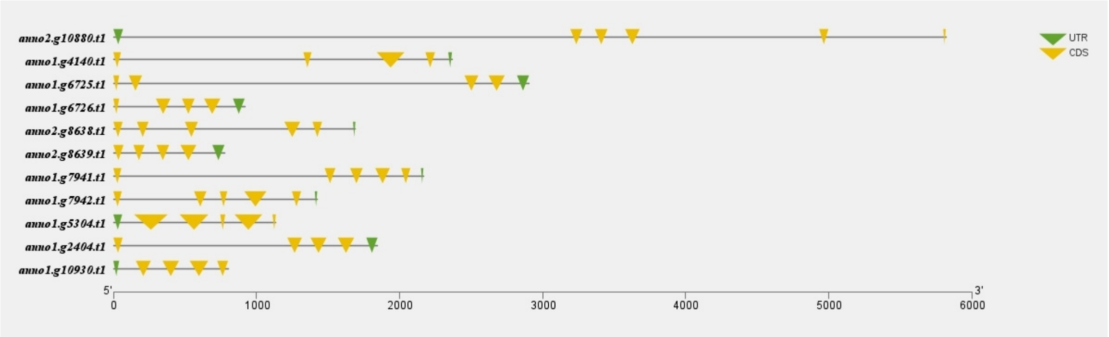

Supplementary Figure S2. Gene structure of OBP family members in *R. brunneum.* Exons are represented by yellow boxes, introns by black lines, and untranslated regions (UTRs) by green boxes. Gene IDs are shown on the left. The scale bar at the bottom indicates the length of the gene (in bp). All OBP genes contain 5–6 exons.

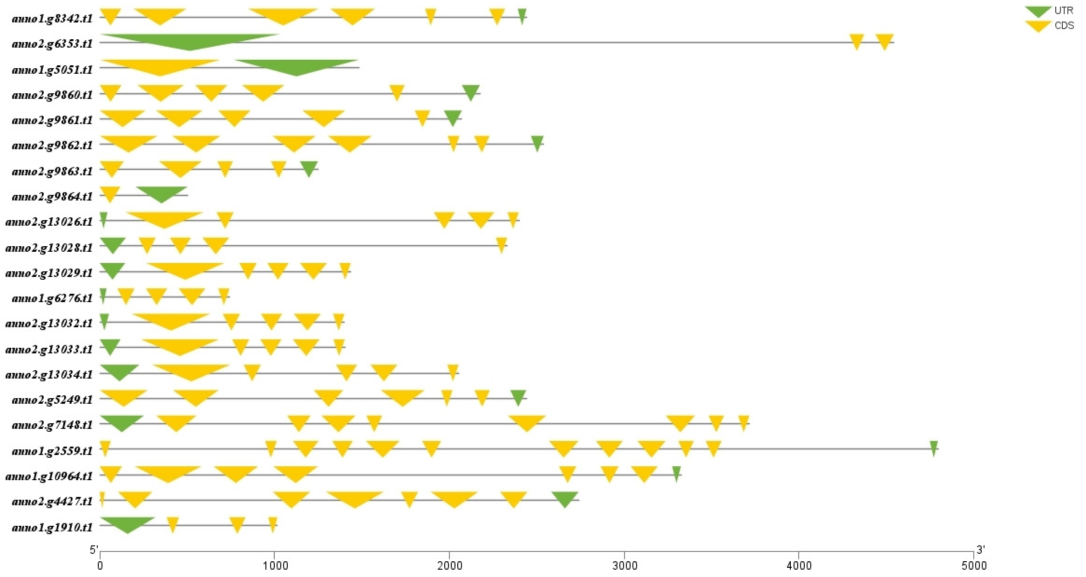

Supplementary Figure S3. Gene structure of GR family members in *R. brunneum.* Exons are represented by yellow boxes, introns by black lines, and untranslated regions (UTRs) by green boxes. Gene IDs are shown on the left. The scale bar at the bottom indicates the length of the gene (in bp). GR genes display relatively compact gene structures with similar exon numbers among members.

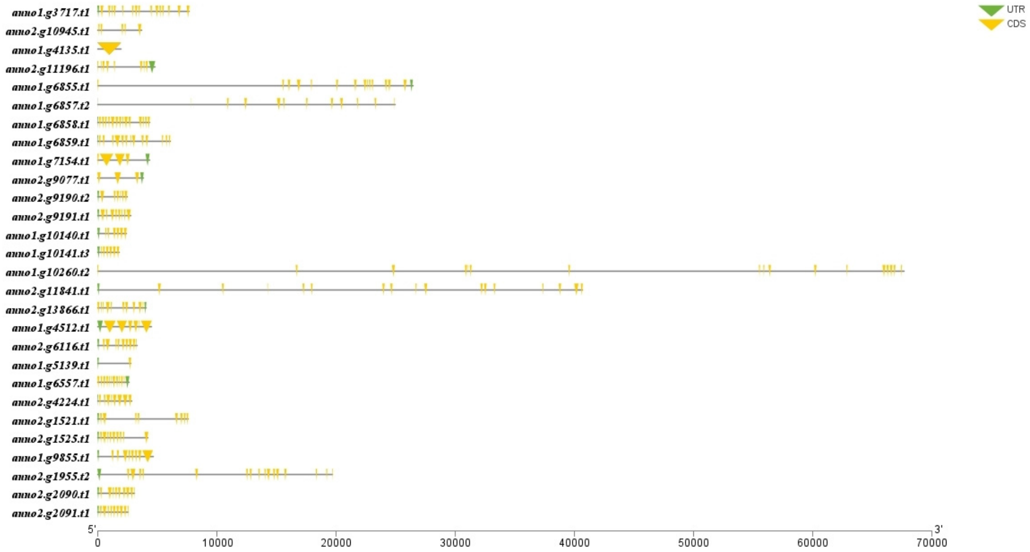

Supplementary Figure S4. Gene structure of IR family members in *R. brunneum.* Exons are represented by yellow boxes, introns by black lines, and untranslated regions (UTRs) by green boxes. Gene IDs are shown on the left. The scale bar at the bottom indicates the length of the gene (in bp). IR genes exhibit more complex gene structures, with some members containing a large number of exons (up to 14).

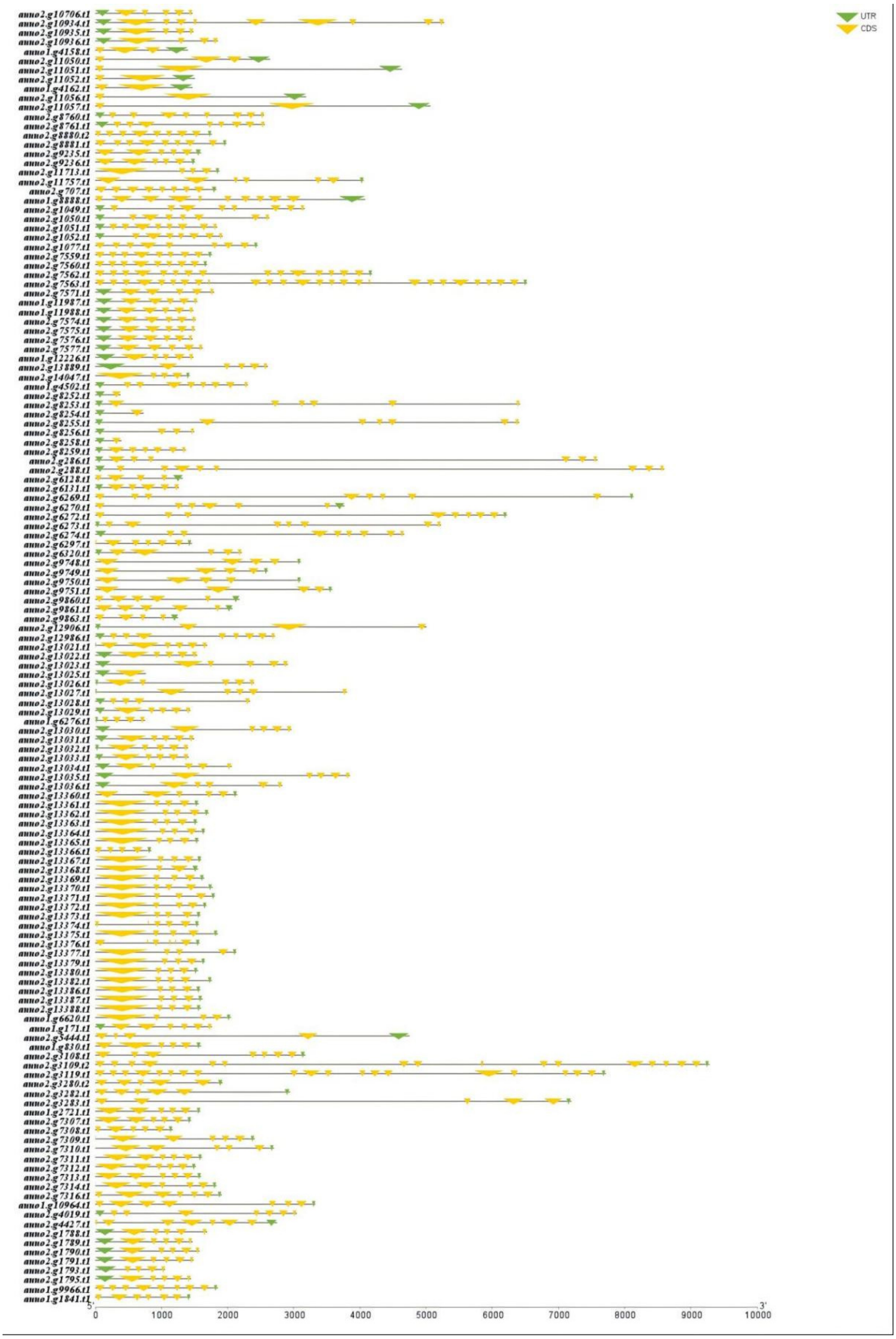

Supplementary Figure S5. Gene structure of OR family members in *R. brunneum.* Exons are represented by yellow boxes, introns by black lines, and untranslated regions (UTRs) by green boxes. Gene IDs are shown on the left. The scale bar at the bottom indicates the length of the gene (in bp). OR genes display comparatively complex gene structures with variable exon numbers.

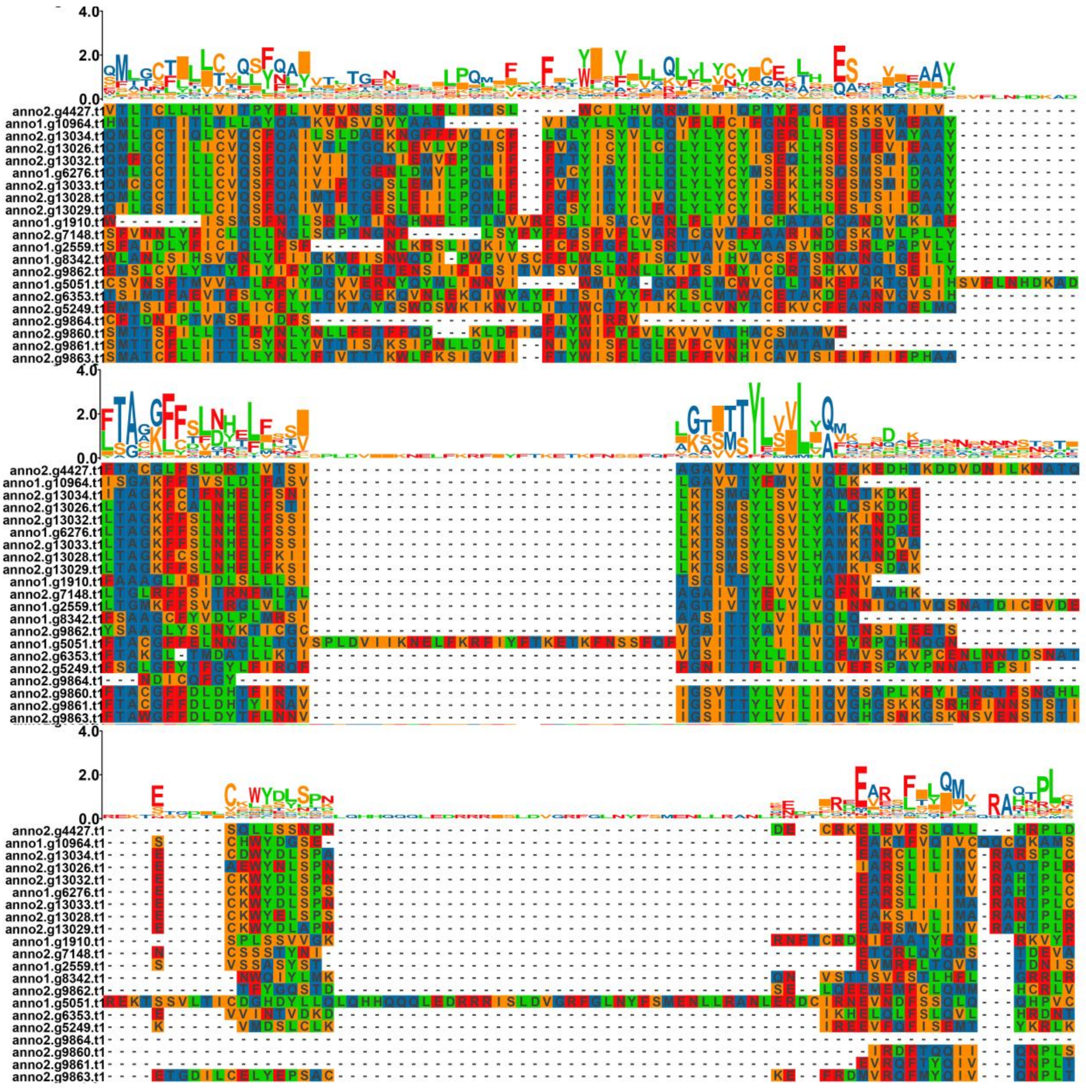

Supplementary Figure S6. Amino acid sequences of GRs family members in *R. brunneum*. The alignment was generated using MAFFT. Conserved residues are shaded according to similarity (dark = identical, light = similar). Gene IDs are shown on the left.

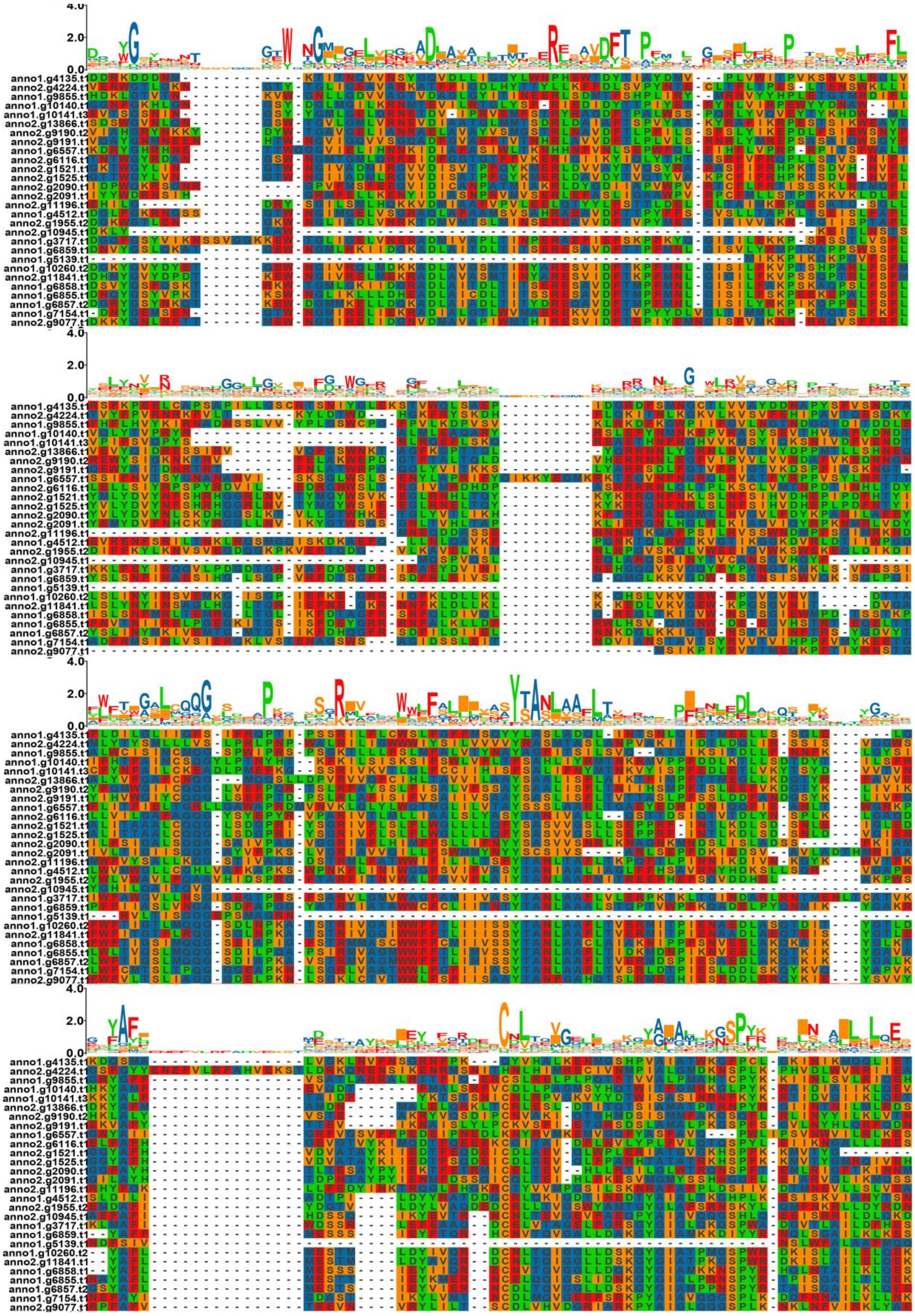

Supplementary Figure S7. Amino acid sequences of IRs family members in *R. brunneum.* The alignment was generated using MAFFT. Conserved residues are shaded according to similarity (dark = identical, light = similar). Gene IDs are shown on the left.

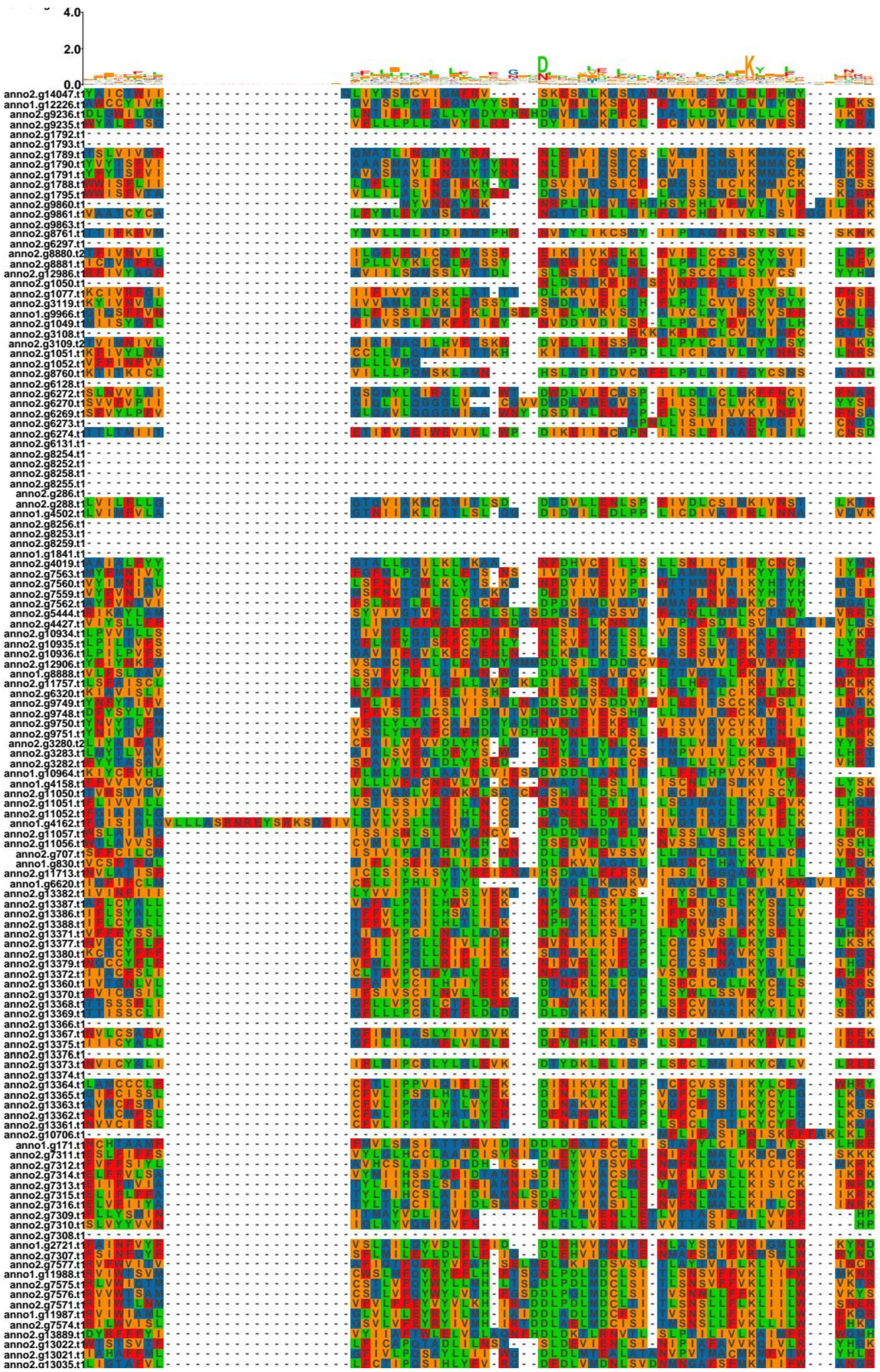
4.0
2.0
0.0

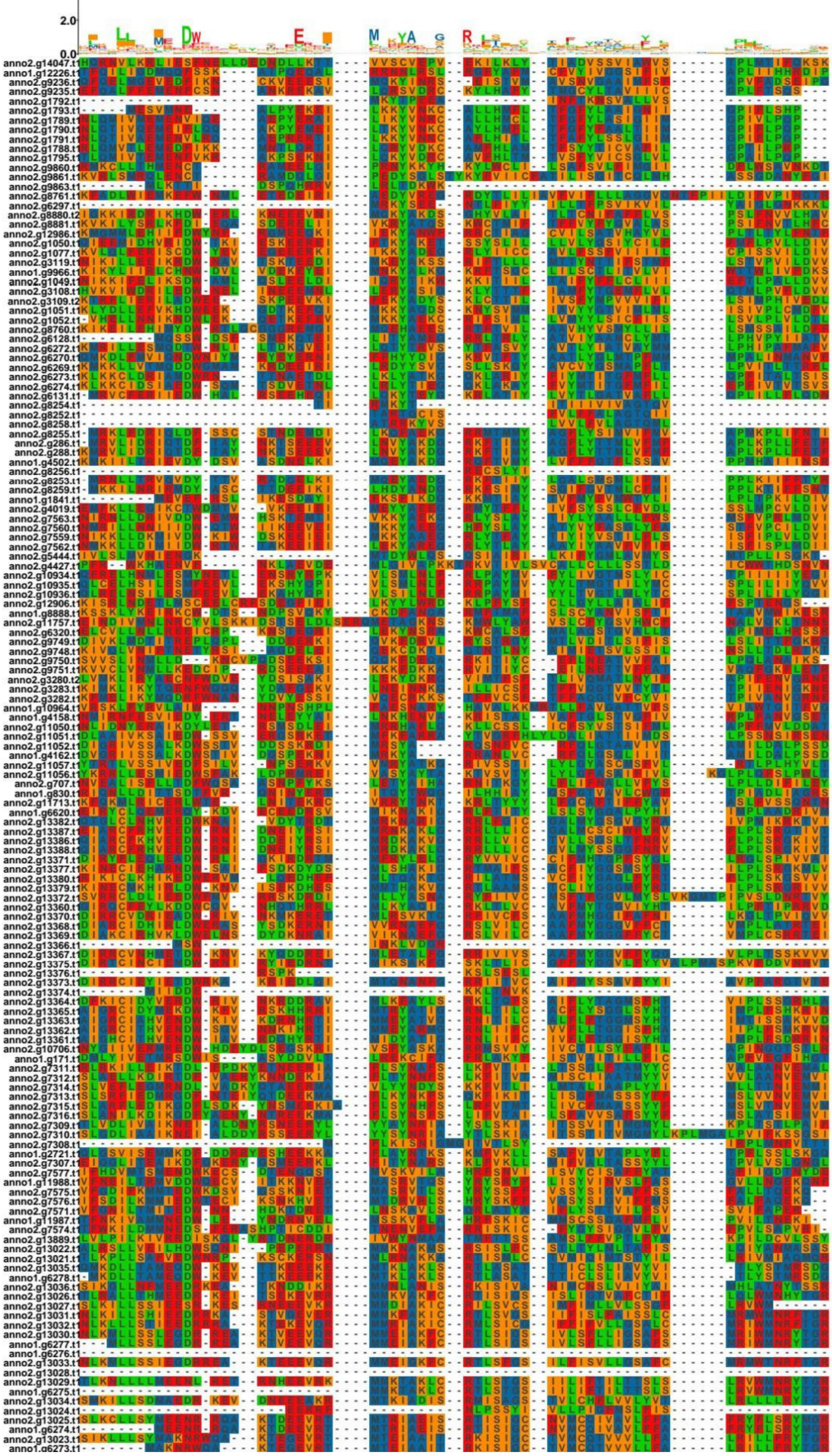

Supplementary Figure S8. Amino acid sequences of ORs family members in *R. brunneum*. The alignment was generated using MAFFT. Conserved residues are shaded according to similarity (dark = identical, light = similar). Gene IDs are shown on the left.

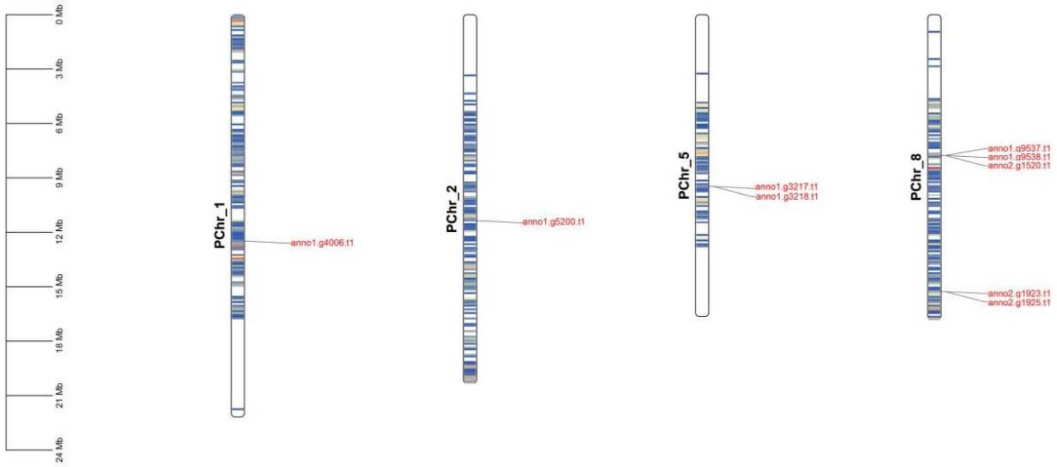

Supplementary Figure S9. Chromosome localization analysis of the CSP gene in *R. brunneum.* The four pseudochromosomes are drawn to scale according to the chromosome-level genome assembly. Each colored rectangle represents gene density, with gene IDs indicate right of the pseudochromosome. The gray lines indicate the locations of genes on pseudochromosomes; on some pseudochromosomes, multiple genes are distributed at the same position, forming tandemly arranged gene clusters.

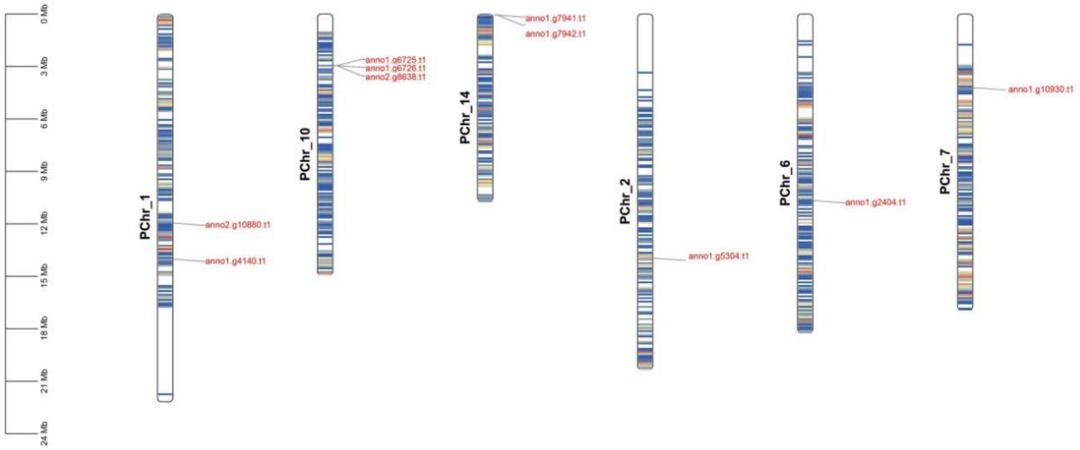

Supplementary Figure S10. Chromosome localization analysis of the OBP gene in *R. brunneum.* The six pseudochromosomes are drawn to scale according to the chromosome-level genome assembly. Each colored rectangle represents gene density, with gene IDs indicate right of the pseudochromosome. The gray lines indicate the locations of genes on pseudochromosomes; on some pseudochromosomes, multiple genes are distributed at the same position, forming tandemly arranged gene clusters.

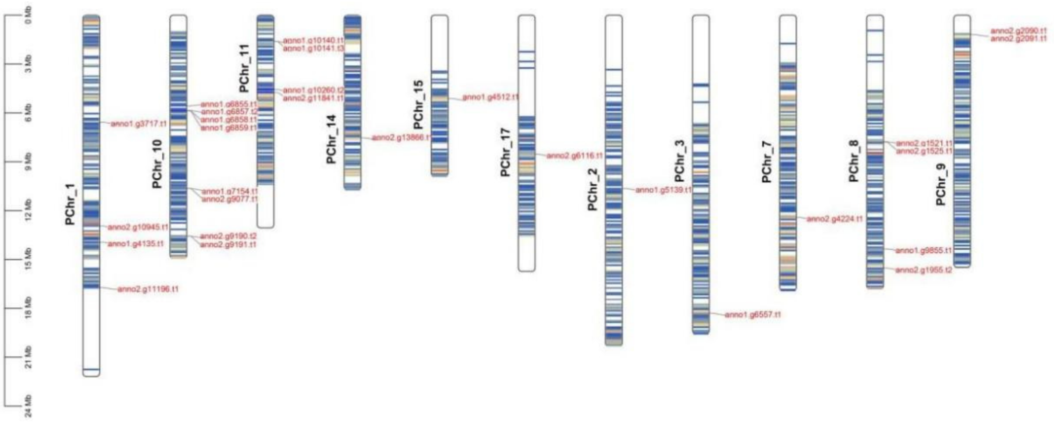

Supplementary Figure S11. Chromosome localization analysis of the IR gene in *R. brunneu.* The eleven pseudochromosomes are drawn to scale according to the chromosome-level genome assembly. Each colored rectangle represents gene density, with gene IDs indicate right of the pseudochromosome. The gray lines indicate the locations of genes on pseudochromosomes; on some pseudochromosomes, multiple genes are distributed at the same position, forming tandemly arranged gene clusters.

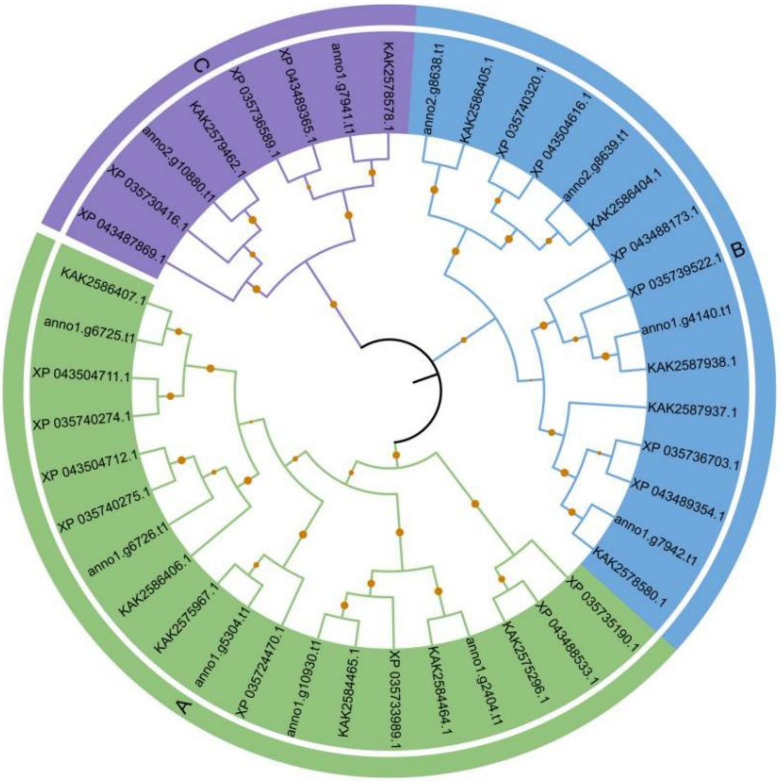

Supplementary Figure S12. Phylogenetic relationships of OBPs among *R. brunneum, O. spinipes, V. mandarinia* and *P. fuscatus* (anno: *R. brunneum*; KAK: *O. spinipes*; XP043: *P. fuscatus*; XP035: *V. mandarinia*). Based on the tree's topology and node support values, the OBP proteins from four species were divided into three major branches, each labeled with distinct colors. The branch classification criteria were as follows: trees constructed using either the neighborjoining (NJ) or maximum likelihood (ML) methods employed a bootstrap value ⩾ 70% as the threshold for node confidence; phylogenetic units corresponding to nodes meeting this support threshold, along with their respective topological structures derived from the phylogenetic tree, were defined as major branches.

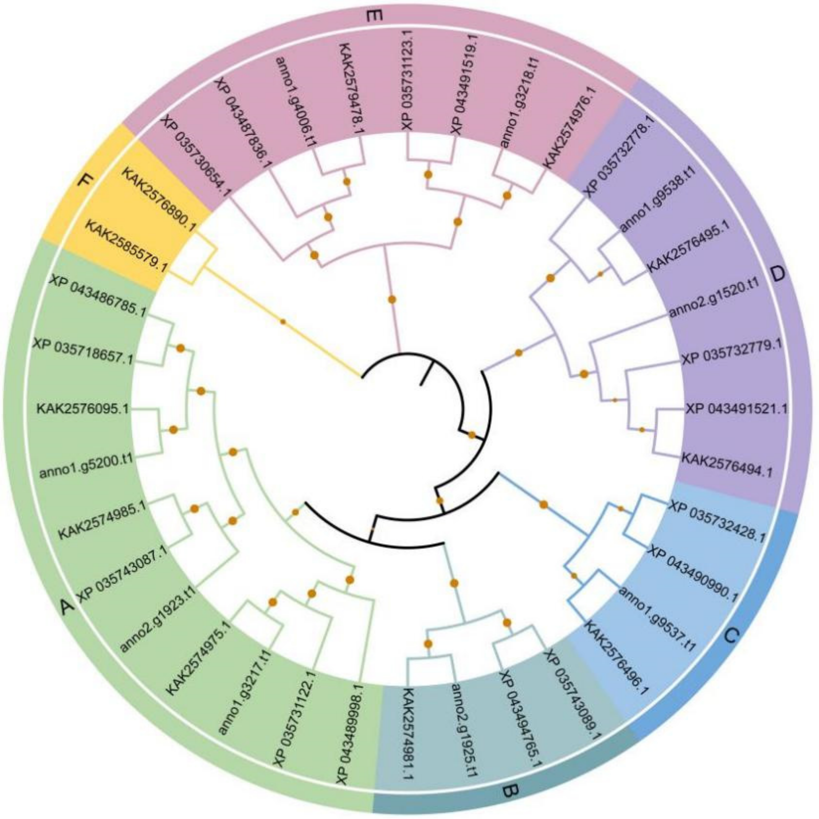

Supplementary Figure S13. Phylogenetic relationships of CSPs among *R. brunneum, O. spinipes, V. mandarinia* and *P. fuscatus* (anno: *R. brunneum*; KAK: *O. spinipes*; XP043: *P. fuscatus*; XP035: *V. mandarinia*). Based on the tree's topology and node support values, the CSP proteins from four species were divided into six major branches, each labeled with distinct colors. The branch classification criteria were as follows: trees constructed using either the neighborjoining (NJ) or maximum likelihood (ML) methods employed a bootstrap value ⩾ 70% as the threshold for node confidence; phylogenetic units corresponding to nodes meeting this support threshold, along with their respective topological structures derived from the phylogenetic tree, were defined as major branches.

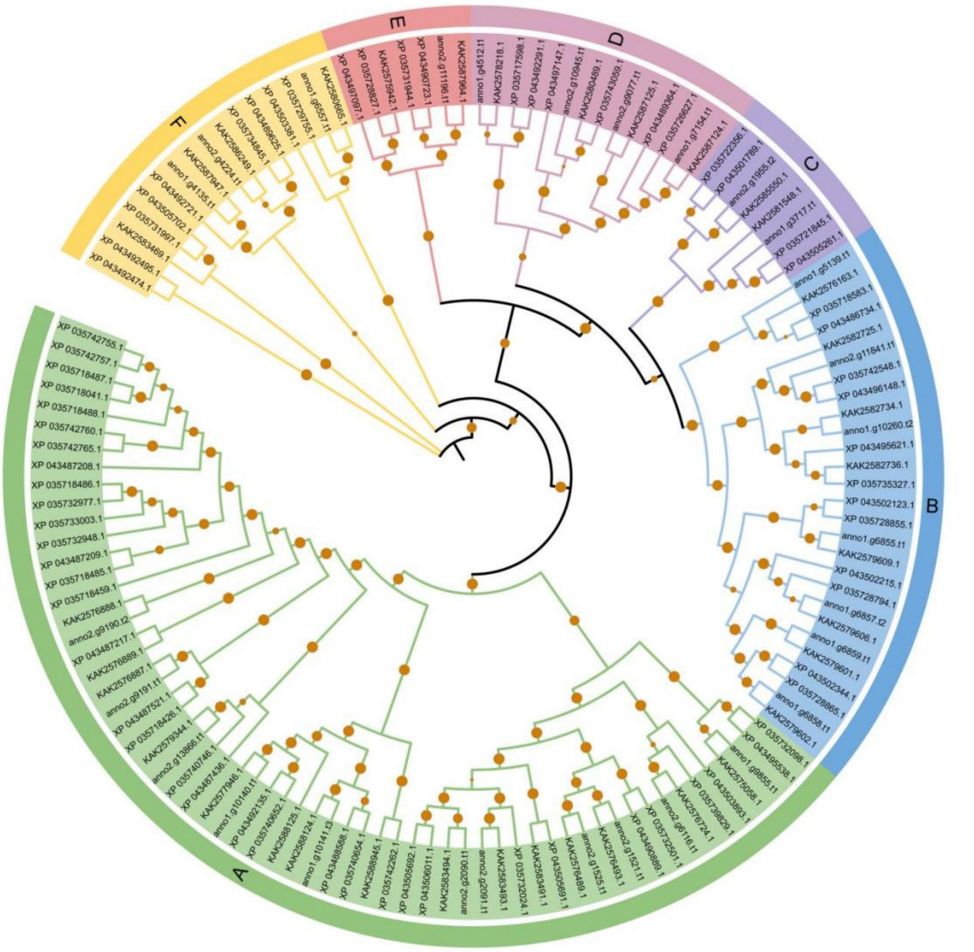

Supplementary Figure S14. Phylogenetic relationships of IRs among *R. brunneum, O. spinipes, V. mandarinia* and *P. fuscatus* (anno: *R. brunneum*; KAK: *O. spinipes*; XP043: *P. fuscatus*; XP035: *V. mandarinia*). Based on the tree's topology and node support values, the IR proteins from four species were divided into six major branches, each labeled with distinct colors. The branch classification criteria were as follows: trees constructed using either the neighborjoining (NJ) or maximum likelihood (ML) methods employed a bootstrap value ⩾ 70% as the threshold for node confidence; phylogenetic units corresponding to nodes meeting this support threshold, along with their respective topological structures derived from the phylogenetic tree, were defined as major branches.

Supplementary Table S1. Summary of physicochemical properties of CSP, OBP, OR, IR, and GR gene families in *R. brunneum*

gene family	Gene ID	Gene length CDS	Amino acid content AA	formula weight MW	isoelectric point	Unstable Coefficient	Position Prediction	Ah
CSP	anno1.g4006.t1	351	116	13049.26	9.35	74.17	extr	-0.27
	anno1.g5200.t1	378	125	14340.49	8.53	26.99	extr	-0.571
	anno1.g3217.t1	372	123	14140.37	9.21	30.48	extr	-0.678
	anno1.g3218.t1	321	106	12435.54	9.83	30.03	extr	-0.382
	anno1.g9537.t1	375	124	14257.52	5.86	35.42	extr	-0.408
	anno1.g9538.t1	372	123	14271.46	5.44	34.59	extr	-0.232
	anno2.g1520.t1	372	123	14398.4	4.98	26.05	extr	-0.466
	anno2.g1923.t1	387	128	14462.73	8.23	48.74	extr	-0.417
	anno2.g1925.t1	423	140	16081.03	4.79	47.3	extr	-0.653
OBP	anno2.g10880.t1	429	142	15715.12	4.36	40.13	extr	-0.028
	anno1.g4140.t1	402	133	15514.04	8.92	55.41	extr	-0.285
	anno1.g6725.t1	435	144	16385.89	4.96	61.57	extr	-0.469
	anno1.g6726.t1	432	143	15551.9	4.97	40.95	extr	-0.117
	anno2.g8638.t1	438	145	16535.26	6.57	24.03	extr	-0.245
	anno2.g8639.t1	438	145	16222.15	5.86	30.55	extr	-0.003
	anno1.g7941.t1	408	135	15420.14	5.51	27.18	extr	-0.066
	anno1.g7942.t1	441	146	16600.51	8.59	41.5	extr	-0.122
	anno1.g5304.t1	756	251	29461.83	5.17	53.59	extr	-1.179
	anno1.g2404.t1	477	158	17528.67	6.71	35.9	extr	-0.106
	anno1.g10930.t1	471	156	17561.2	5.09	33.19	extr	-0.031
IR	anno1.g3717.t1	2865	954	106138.43	6.14	37.78	plas	-0.129
	anno2.g10945.t1	855	284	32597.01	6.55	49.66	plas	-0.542
	anno1.g4135.t1	2001	666	75860.05	4.61	42.23	plas	0.134
	anno2.g11196.t1	1701	566	64718.89	9.19	44.33	plas	-0.031
	anno1.g6855.t1	2787	928	104098.82	5.2	40.03	plas	-0.179
	anno1.g6857.t2	1743	580	65072.69	6.69	32.99	plas	-0.153
	anno1.g6858.t1	2694	897	101408.68	5.49	36.08	plas	-0.098
	anno1.g6859.t1	2610	869	98465.41	5.06	33.24	plas	-0.136
	anno1.g7154.t1	2790	929	106399.1	5.32	42.05	plas	-0.253
	anno2.g9077.t1	1443	480	56402.98	8.28	40.48	plas	-0.188
	anno2.g9190.t2	1353	450	51649.6	9.48	32.68	plas	0.132
	anno2.g9191.t1	1953	650	73419.96	7.99	37.81	plas	0.146
	anno1.g10140.t1	1431	476	55901.22	9.35	33.12	E.R	-0.036
	anno1.g10141.t3	1380	459	53039.57	9.21	36.6	plas	0.013
	anno1.g10260.t2	2724	907	103553.99	5.69	33.97	plas	-0.124
	anno2.g11841.t1	3204	1067	119865.26	5.77	49.31	plas	-0.27
	anno2.g13866.t1	1914	637	73133.68	6.73	32.46	plas	0.057

Supplementary Table S1 (continued) Summary of physicochemical properties of CSP, OBP, OR, IR, and GR gene families in *R. brunneum*

gene family	Gene ID	Gene length CDS	Amino acid content AA	formula weight MW	isoelectric point	Unstable Coefficient	Position Prediction	Ah
	anno1.g4512.t1	4068	1355	150964.6	9.67	51.49	plas	-0.242
	anno2.g6116.t1	1974	657	75943.47	6.82	45.13	plas	-0.114
	anno1.g5139.t1	345	114	12637.7	9.14	40.46	plas	0.122
	anno1.g6557.t1	1905	634	73962.88	7.6	47.4	plas	-0.179
	anno2.g4224.t1	2112	703	82063.26	9.06	39.83	plas	-0.075
	anno2.g1521.t1	1680	559	64899.14	8.26	37.28	plas	-0.01
	anno2.g1525.t1	1962	653	75960.45	6.45	45.55	plas	-0.102
	sanno1.g9855.t1	2676	891	101183.57	8.88	39.03	plas	-0.315
	anno2.g1955.t2	3237	1078	122177.18	6.41	36.27	plas	-0.138
	anno2.g2090.t1	1908	635	72648.79	9.3	44.63	plas	0.113
	anno2.g2091.t1	1905	634	72866.54	9.07	39.99	plas	-0.012
GR	anno1.g8342.t1	1302	433	50201.09	8.88	43.35	plas	0.332
	anno2.g6353.t1	1227	408	47259.61	8.77	32.51	plas	0.239
	anno1.g5051.t1	1416	471	54759.35	9.09	36.57	plas	0.291
	anno2.g9860.t1	1020	339	39881.09	9.21	39.89	mito	0.282
	anno2.g9861.t1	1161	386	44148.72	8.95	41.93	plas	0.324
	anno2.g9862.t1	1347	448	52138.97	8.76	52.26	plas	0.192
	anno2.g9863.t1	672	223	25789.02	8.82	38.64	cyto	0.161
	anno2.g9864.t1	420	139	16313.16	6.94	50.99	plas	0.503
	anno2.g13026.t1	942	313	35552.71	7.54	43.45	plas	0.337
	anno2.g13028.t1	597	198	22925.23	7.62	37.75	extr	0.419
	anno2.g13029.t1	1044	347	39536.79	8.72	33.04	plas	0.431
	anno1.g6276.t1	486	161	18422.88	5.43	56.03	plas	0.634
	anno2.g13032.t1	951	316	36745.69	7.53	42.96	plas	0.467
	anno2.g13033.t1	1017	338	38909.08	6.39	41.05	plas	0.49
	anno2.g13034.t1	1125	374	43319.1	8.5	38.25	plas	0.291
	anno2.g5249.t1	1212	403	47136.74	8.42	38.53	plas	0.442
	anno2.g7148.t1	1458	485	54749.37	9.64	43.14	plas	0.196
	anno1.g2559.t1	1425	474	53355.09	8.91	36.23	plas	0.313
	anno1.g10964.t1	1437	478	53797.94	8.06	32.24	extr	0.295
	anno2.g4427.t1	1473	490	55762.24	8.95	35.95	plas	0.095
	anno1.g1910.t1	543	180	20009.43	7.63	37.72	plas	0.535
OR	anno2.g10706.t1	978	325	37034.3	8.72	48.46	plas	0.346
	anno2.g10934.t1	2394	797	92343.7	8.88	40.03	plas	0.485
	anno2.g10935.t1	1179	392	45705.02	6.14	46.82	plas	0.46
	anno2.g10936.t1	1185	394	45743.45	8.42	44.56	plas	0.495
	anno1.g4158.t1	1200	399	45300.57	6.65	35.4	plas	0.505

Supplementary Table S1 (continued) Summary of physicochemical properties of CSP, OBP, OR, IR, and GR gene families in *R. brunneum*

gene family	Gene ID	Gene length CDS	Amino acid content AA	formula weight MW	isoelectric point	Unstable Coefficient	Position Prediction	Ah
	anno2.g11050.t1	1188	395	44899.92	7.4	32.05	plas	0.564
	anno2.g11051.t1	1191	396	44974.73	7.53	39.28	plas	0.359
	anno2.g11052.t1	1197	398	44776.05	5.56	34.59	plas	0.38
	anno1.g4162.t1	1254	417	47260.04	7.56	35.57	plas	0.29
	anno2.g11056.t1	1212	403	45710.66	7.94	41.42	plas	0.362
	anno2.g11057.t1	1200	399	45403.17	6.66	38.57	plas	0.367
	anno2.g8760.t1	1179	392	45023.59	6.01	43.21	plas	0.514
	anno2.g8761.t1	1227	408	47500.34	9.07	41.46	plas	0.4
	anno2.g8880.t2	1152	383	44741.22	8.13	35.04	plas	0.583
	anno2.g8881.t1	1203	400	47132.9	9.05	38.84	plas	0.429
	anno2.g9235.t1	1137	378	42978.73	9.03	39.71	plas	0.41
	anno2.g9236.t1	1119	372	42581.35	7.03	33.45	mito	0.362
	anno2.g11713.t1	1206	401	45347.9	6.94	34.86	plas	0.374
	anno2.g11757.t1	1323	440	50609.34	5.88	39.92	plas	0.274
	anno2.g707.t1	1200	399	45700.75	6.54	49.83	plas	0.483
	anno1.g8888.t1	2292	763	87370.75	9.22	36.49	plas	0.203
	anno2.g1049.t1	1185	394	45606.18	6.66	29.66	plas	0.645
	anno2.g1050.t1	1065	354	41587.96	6.32	47.67	plas	0.456
	anno2.g1051.t1	1167	388	45291.85	8.83	38.06	plas	0.411
	anno2.g1052.t1	1062	353	41163.22	8.84	31.43	plas	0.525
	anno2.g1077.t1	1185	394	45831.96	8.69	36.21	plas	0.584
	anno2.g7559.t1	1167	388	44905.89	9.01	37.08	plas	0.38
	anno2.g7560.t1	1173	390	45547.46	8.34	46.11	plas	0.351
	anno2.g7562.t1	2157	718	82839.52	7.8	40.21	nucl	0.43
	anno2.g7563.t1	3537	1178	136409.56	8.15	41.86	plas	0.371
	anno2.g7571.t1	1119	372	42364.52	5.91	34.93	plas	0.412
	anno1.g11987.t1	1131	376	43135.18	5.85	39.39	plas	0.553
	anno1.g11988.t1	1134	377	43500.1	8.67	45.44	plas	0.379
	anno2.g7574.t1	1131	376	42869.57	5.85	38.64	plas	0.523
	anno2.g7575.t1	1131	376	42984.58	8.11	44.76	plas	0.463
	anno2.g7576.t1	1131	376	43223.11	8.43	36.08	plas	0.479
	anno2.g7577.t1	1155	384	43276.57	6	39.05	plas	0.745
	anno1.g12226.t1	1131	376	43016.32	9.23	32.14	plas	0.635
	anno2.g13889.t1	1167	388	44441.32	8.48	36.63	plas	0.426
	anno2.g14047.t1	1161	386	44203.96	7.96	33.02	plas	0.385
	anno1.g4502.t1	1167	388	44690.09	6.12	42.23	plas	0.331
	anno2.g8252.t1	270	89	9868.82	9.24	21.11	plas	0.639

Supplementary Table S1 (continued) Summary of physicochemical properties of CSP, OBP, OR, IR, and GR gene families in *R. brunneum*

gene family	Gene ID	Gene length CDS	Amino acid content AA	formula weight MW	isoelectric point	Unstable Coefficient	Position Prediction	Ah
	anno2.g8253.t1	903	300	34785.53	7.68	27.08	plas	0.233
	anno2.g8254.t1	336	111	12900.38	8.73	17.2	mito	0.612
	anno2.g8255.t1	906	301	34902.59	6.79	52.8	plas	0.135
	anno2.g8256.t1	462	153	18012.36	9.72	47.62	plas	0.32
	anno2.g8258.t1	276	91	10326.38	9.93	24.5	mito	0.422
	anno2.g8259.t1	903	300	34848.09	9.19	36.3	plas	0.297
	anno2.g286.t1	903	300	34609.31	6.36	39.57	plas	0.273
	anno2.g288.t1	1170	389	44781.32	6.41	43.36	plas	0.267
	anno2.g6128.t1	714	237	27203.48	5.39	41.94	plas	0.189
	anno2.g6131.t1	924	307	35437.94	8.36	51.66	plas	0.19
	anno2.g6269.t1	1188	395	45207.92	8.45	39.84	plas	0.279
	anno2.g6270.t1	1002	333	38872.28	4.97	31.4	plas	0.218
	anno2.g6272.t1	1179	392	45237.99	6.17	44.88	plas	0.371
	anno2.g6273.t1	978	325	37424.76	6.65	39.15	plas	0.287
	anno2.g6274.t1	1197	398	45737.52	6.16	41.72	plas	0.283
	anno2.g6297.t1	828	275	32147.43	8.6	38.4	plas	0.539
	anno2.g6320.t1	1125	374	43611.57	8.38	50.44	plas	0.471
	anno2.g9748.t1	1110	369	42436.82	5.64	36.39	plas	0.501
	anno2.g9749.t1	1119	372	43482.59	8.7	33	plas	0.467
	anno2.g9750.t1	1161	386	44893.77	7.03	41.76	plas	0.418
	anno2.g9751.t1	1164	387	44878.18	9.1	36.63	plas	0.354
	anno2.g9860.t1	1020	339	39881.09	9.21	39.89	mito	0.282
	anno2.g9861.t1	1161	386	44148.72	8.95	41.93	plas	0.324
	anno2.g9863.t1	672	223	25789.02	8.82	38.64	cyto	0.161
	anno2.g12906.t1	1068	355	40826.91	6.97	36.57	plas	0.338
	anno2.g12986.t1	1179	392	46001.69	8.51	41.9	plas	0.548
	anno2.g13021.t1	1221	406	46432.48	8.19	43.4	plas	0.195
	anno2.g13022.t1	1197	398	45140.3	6.45	45.88	plas	0.505
	anno2.g13023.t1	1125	374	42774.72	8.53	32.84	plas	0.488
	anno1.g6273.t1	441	146	16546.45	9.14	29.35	plas	0.338
	anno2.g13024.t1	237	78	8950.51	6.04	43.79	plas	0.483
	anno2.g13025.t1	684	227	26118.57	8.95	38.45	plas	0.306
	anno1.g6274.t1	432	143	16468.09	9.1	38.64	plas	0.071
	anno2.g13026.t1	942	313	35552.71	7.54	43.45	plas	0.337
	anno2.g13027.t1	936	311	36008.43	6.09	51.71	plas	0.368
	anno2.g13028.t1	597	198	22925.23	7.62	37.75	extr	0.419
	anno2.g13029.t1	1044	347	39536.79	8.72	33.04	plas	0.431

Supplementary Table S1 (continued) Summary of physicochemical properties of CSP, OBP, OR, IR, and GR gene families in *R. brunneum*

gene family	Gene ID	Gene length CDS	Amino acid content AA	formula weight MW	isoelectric point	Unstable Coefficient	Position Prediction	Ah
	anno1.g6275.t1	399	132	14991.64	9.66	34.75	extr	0.132
	anno1.g6276.t1	486	161	18422.88	5.43	56.03	plas	0.634
	anno2.g13030.t1	1125	374	42839.83	7.54	39.87	plas	0.529
	anno1.g6277.t1	447	148	17064.05	8.68	29.17	plas	0.309
	anno2.g13031.t1	1089	362	41499.35	8.58	36.71	plas	0.56
	anno2.g13032.t1	951	316	36745.69	7.53	42.96	plas	0.467
	anno2.g13033.t1	1017	338	38909.08	6.39	41.05	plas	0.49
	anno2.g13034.t1	1125	374	43319.1	8.5	38.25	plas	0.291
	anno2.g13035.t1	1176	391	45440.45	6.02	42.56	plas	0.283
	anno1.g6278.t1	456	151	17505.38	6.89	31.33	plas	0.025
	anno2.g13036.t1	1119	372	42984.12	5.4	41.04	plas	0.244
	anno2.g13360.t1	1224	407	46358.94	8.98	41.03	plas	0.355
	anno2.g13361.t1	1200	399	45607.02	7.48	34.32	plas	0.442
	anno2.g13362.t1	1206	401	45879.69	9.01	24.71	plas	0.47
	anno2.g13363.t1	1206	401	45374.55	7.06	22.84	plas	0.513
	anno2.g13364.t1	1242	413	47668.26	7.75	34.51	plas	0.422
	anno2.g13365.t1	1236	411	47267.02	9.09	35.88	plas	0.339
	anno2.g13366.t1	522	173	19758.26	7.64	39.79	plas	0.42
	anno2.g13367.t1	1218	405	45593.81	5.78	37.66	plas	0.505
	anno2.g13368.t1	1257	418	46962.39	6.65	43.42	plas	0.452
	anno2.g13369.t1	1224	407	46310.81	8.02	47.98	plas	0.401
	anno2.g13370.t1	1233	410	46541	9.07	43.13	plas	0.474
	anno2.g13371.t1	1215	404	46449.14	8.29	42.96	plas	0.486
	anno2.g13372.t1	1224	407	46587.12	6.98	48.3	plas	0.476
	anno2.g13373.t1	1230	409	46600.67	8.08	50.16	plas	0.337
	anno2.g13374.t1	498	165	19017.43	5.01	32.99	plas	0.332
	anno2.g13375.t1	1212	403	45912.41	6.45	40.56	plas	0.521
	anno2.g13376.t1	531	176	19996.26	7.75	46.78	cyto	0.002
	anno2.g13377.t1	1224	407	46425.92	8.76	43.41	plas	0.386
	anno2.g13379.t1	1230	409	46652.3	8.61	55.97	plas	0.341
	anno2.g13380.t1	1248	415	47228.09	8.97	45.68	plas	0.296
	anno2.g13382.t1	1209	402	45581.24	9.17	35.32	plas	0.431
	anno2.g13386.t1	1230	409	46281.98	8.95	35.86	plas	0.476
	anno2.g13387.t1	1227	408	46703.12	8.7	42.51	plas	0.353
	anno2.g13388.t1	1230	409	46692.24	8.98	45.35	plas	0.351
	anno1.g6620.t1	1218	405	46868.51	5.63	39.2	plas	0.445
	anno1.g171.t1	1164	387	43193.49	6.25	37.77	plas	0.466

Supplementary Table S1 (continued) Summary of physicochemical properties of CSP, OBP, OR, IR, and GR gene families in *R. brunneum*

gene family	Gene ID	Gene length CDS	Amino acid content AA	formula weight MW	isoelectric point	Unstable Coefficient	Position Prediction	Ah
	anno2.g5444.t1	1074	357	40509.94	5.78	48.41	plas	0.543
	anno1.g830.t1	1218	405	46009.13	8.96	40.88	plas	0.426
	anno2.g3108.t1	1161	386	44763.6	8.26	36.69	cyto	0.109
	anno2.g3109.t2	2208	735	85715.93	8.53	42.68	plas	0.51
	anno2.g3119.t1	2958	985	113757.15	8.04	32.82	plas	0.574
	anno2.g3280.t2	1218	405	46872.59	8.5	43.07	plas	0.492
	anno2.g3282.t1	1233	410	47499.41	6.7	43.26	plas	0.362
	anno2.g3283.t1	1197	398	45709.81	6.39	30.78	plas	0.467
	anno1.g2721.t1	1161	386	44389.16	8.3	41.72	plas	0.366
	anno2.g7307.t1	1119	372	42378.22	7.96	45.42	plas	0.557
	anno2.g7308.t1	792	263	30353.22	8.86	46.11	plas	0.572
	anno2.g7309.t1	1182	393	45019.3	8.6	26.75	plas	0.468
	anno2.g7310.t1	1194	397	45215.43	8.48	31.74	plas	0.493
	anno2.g7311.t1	1158	385	44067.08	8.03	42.09	plas	0.41
	anno2.g7312.t1	1191	396	45299.84	8.39	42.44	plas	0.467
	anno2.g7313.t1	1104	367	42360.32	6.39	49.34	plas	0.591
	anno2.g7314.t1	1164	387	44097.85	8.05	42.68	plas	0.657
	anno2.g7315.t1	1158	385	44309.53	8.06	38.88	plas	0.497
	anno2.g7316.t1	1260	419	47653.19	8.43	44.29	plas	0.415
	anno1.g10964.t1	1437	478	53797.94	8.06	32.24	extr	0.295
	anno2.g4019.t1	1050	349	40799.74	7.86	39.16	plas	0.625
	anno2.g4427.t1	1473	490	55762.24	8.95	35.95	plas	0.095
	anno2.g1788.t1	1125	374	42380.36	8.87	42.18	plas	0.586
	anno2.g1789.t1	1110	369	41592.39	8.6	38.57	plas	0.54
	anno2.g1790.t1	1110	369	41854.31	6.95	42.73	plas	0.447
	anno2.g1791.t1	1110	369	41960.56	9.01	43.9	plas	0.417
	anno2.g1792.t1	303	100	11355.31	7.71	23.81	extr	0.305
	anno2.g1793.t1	729	242	27305.2	6.58	45.87	plas	0.664
	anno2.g1795.t1	1116	371	41963.19	9.74	41.2	plas	0.546
	anno1.g9966.t1	1182	393	45706.42	8.59	41.43	plas	0.612
	anno1.g1841.t1	906	301	34707.94	9.39	37.68	cyto	0.288
